# Venus looks different from day to night across wavelengths: morphology from Akatsuki multispectral images

**DOI:** 10.1186/s40623-018-0789-5

**Published:** 2018-02-12

**Authors:** Sanjay S. Limaye, Shigeto Watanabe, Atsushi Yamazaki, Manabu Yamada, Takehiko Satoh, Takao M. Sato, Masato Nakamura, Makoto Taguchi, Tetsuya Fukuhara, Takeshi Imamura, Toru Kouyama, Yeon Joo Lee, Takeshi Horinouchi, Javier Peralta, Naomoto Iwagami, George L. Hashimoto, Seiko Takagi, Shoko Ohtsuki, Shin-ya Murakami, Yukio Yamamoto, Kazunori Ogohara, Hiroki Ando, Ko-ichiro Sugiyama, Nobuaki Ishii, Takumi Abe, Chikako Hirose, Makoto Suzuki, Naru Hirata, Eliot F. Young, Adriana C. Ocampo

**Affiliations:** 1 Space Science and Engineering Center, University of Wisconsin, Madison, WI 53706 USA; 2 Space Information Center, Hokkaido Information University, Ebetsu, Hokkaido 069-8585 Japan; 3 Institute of Space and Astronautical Science, Japan Aerospace Exploration Agency, 3-1-1, Yoshinodai, Chuo-ku, Sagamihara, 252-5210 Japan; 4 Planetary Exploration Research Center, Chiba Institute of Technology, 2-17-1, Tsudanuma, Narashino, Chiba 275-0016 Japan; 5 College of Science, Rikkyo University, 3-34-1 Nishi-Ikebukuro, Toshima-ku, Tokyo, 171-8501 Japan; 6 Department of Complexity Science and Engineering, Graduate School of Frontier Sciences, The University of Tokyo, Kiban-tou 4H7, 5-1-5 Kashiwanoha, Kashiwa, Chiba 277-8561 Japan; 7 Artificial Intelligence Research Center, National Institute of Advanced Industrial Science and Technology, Tokyo, Japan; 8 Present Address: Department of Complexity Science and Engineering, Graduate School of Frontier Sciences, The University of Tokyo, Kiban-tou 4E5, 5-1-5 Kashiwanoha, Kashiwa, Chiba 277-8561 Japan; 9 Faculty of Environmental Earth Science, Hokkaido University, N10W5, Sapporo, Hokkaido 060-0810 Japan; 10 School of Commerce, Senshu University, 2-1-1 Higashimita, Tama-ku, Kawasaki, Kanagawa 214-8580 Japan; 11 Department of Earth Sciences, Okayama University, 3-1-1 Tsushimanaka, Kita-ku, Okayama, 700-8530 Japan; 12 Tokai University, Research and Information Center, 4-1-1 Kitakaname, Hiratsuka-shi, Kanagawa 259-1292 Japan; 13 School of Engineering, University of Shiga Prefecture, Hikone, Japan; 14 Faculty of Science, Kyoto Sangyo University, Motoyama, Kamigamo, Kita-ku, Kyoto-City, 603-8555 Japan; 15 Department of Information Engineering, National Institute of Technology, Matsue College, 14-4 Nishi-Ikuma, Matsue, Shimane 690-8518 Japan; 16 ARC-Space, CAIST, The University of Aizu, 90 Kami-Iawase, Tsuruga, Ikki-machi, Aizu-Wakamatsu, Fukushima 965-8580 Japan; 17 Southwest Research Institute, 1050 Walnut St., Suite 300, Boulder, CO 80302 USA; 18 NASA Headquarters, 300 E Street SW, Washington, DC 20546 USA

**Keywords:** Venus clouds, Morphology, Day, Night, Ultraviolet, Near infrared

## Abstract

Since insertion into orbit on December 7, 2015, the Akatsuki orbiter has returned global images of Venus from its four imaging cameras at eleven discrete wavelengths from ultraviolet (283 and 365 nm) and near infrared (0.9–2.3 µm), to the thermal infrared (8–12 µm) from a near-equatorial orbit. The Venus Express and Pioneer Venus Orbiter missions have also monitored the planet for long periods but from polar or near-polar orbits. The wavelength coverage and views of the planet also differ for all three missions. In reflected light, the images reveal features seen near the cloud tops (~ 70 km altitude), whereas in the near-infrared images of the nightside, features seen are at mid- to lower cloud levels (~ 48–60 km altitude). The dayside cloud cover imaged at the ultraviolet wavelengths shows morphologies similar to what was observed from Mariner 10, Pioneer Venus, Galileo, Venus Express and MESSENGER. The daytime images at 0.9 and 2.02 µm also reveal some interesting features which bear similarity to the ultraviolet images. The nighttime images at 1.74, 2.26 and 2.32 µm and at 8–12 µm reveal features not seen before and show new details of the nightside including narrow wavy ribbons, curved string-like features, long-scale waves, long dark streaks, isolated bright spots, sharp boundaries and even mesoscale vortices. Some features previously seen such as circum-equatorial belts (CEBs) and occasional areal brightenings at ultraviolet (seen in Venus Express observations) of the cloud cover at ultraviolet wavelengths have not been observed thus far. Evidence for the hemispheric vortex organization of the global circulation can be seen at all wavelengths on the day- and nightsides. Akatsuki images reveal new and puzzling morphology of the complex nightside cloud cover. The cloud morphologies provide some clues to the processes occurring in the atmosphere and are thus, a key diagnostic tool when quantitative dynamical analysis is not feasible due to insufficient information.
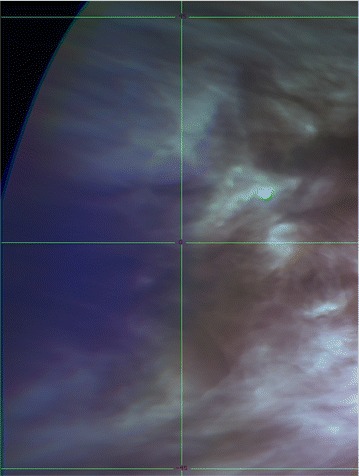

## Akatsuki observations

The Akatsuki orbiter was originally planned to be inserted into Venus orbit on December 7, 2010, after a successful launch on May 21, 2010, and after a short cruise to Venus, into a near-equatorial 30-h elliptic orbit moving in the same direction as the planet to study the superrotation (Nakamura et al. 2011). However, a malfunction during the procedure led to the spacecraft missing orbital insertion and ending up in an ~ 205-day orbit around the Sun (Nakamura et al. 2016). The orbit was altered to a 199-day period by November 2011, while the spacecraft navigation team found a solution to encounter Venus again and to attempt orbit insertion. Orbit insertion was achieved on December 7, 2015, thanks to the careful work of the navigation team by using only the attitude control thrusters, achieving a rare reprieve for a planetary mission. The operational orbit achieved has a similar near-equatorial inclination and eccentricity, but a much longer period (10.5 days). Despite the unplanned extended exposure to much harsher solar thermal conditions during the eleven perihelion passages, the cameras are performing well, while some effects on the readout electronics of the four quadrants of the IR1 camera (Iwagami et al. 2018) and the IR2 camera (Satoh et al. 2017) were discovered. The impact was that the quadrant boundaries were visible and in IR1, the pixels along the column direction above and below the planet’s image on the CCD were filled occasionally with constant values. Both IR1 and IR2 images also showed lines at the internal boundaries of the quadrants (due to slightly different gains of the separate readout electronics for the four quadrants). These anomalies were not present in the laboratory images and in the images acquired during the first orbit insertion attempt in 2010. Image data processing and calibration procedures have been developed and adapted to remove these effects. The Akatsuki orbiter is providing equatorial, global images of Venus from its four cameras with spatial resolution comparable to or better than those obtained from the Venus Monitoring Camera (Markiewicz 2007) on Venus Express. The VIRTIS instrument on Venus Express imaged Venus in both reflected and emitted radiation between 0.28 and 5 µm (Drossart 2007; Piccioni et al. 2007b) and mostly covered portions of the southern hemisphere. The Akatsuki images provide excellent symmetric views of Venus about its equator on day- and nightsides with good image quality spatial resolution from the 1024 × 1024 pixel detectors of the UVI, IR1 and IR2 cameras. The thermal infrared camera (LIR) uses a smaller detector and yields smaller sized images of Venus (~ 18 pixel diameter at apoapsis). Imaging in reflected light is performed in portions of the orbit when the phase angle is appropriate with imaging at roughly 2-h intervals. Imaging of the nightside of Venus is limited to portions of the orbit when the angular separation between the Sun and Venus is larger than 26.5° to avoid stray light. Also, pointing is adjusted to keep as much of the saturated dayside portion of the planet out of the field of view of the IR2 and IR1 cameras as possible to minimize data contamination by the bright dayside portion of the planet. Additionally, data communication to the 64 m Usuda Deep Space Station near Nagano, Japan, occurs daily for about 8 h (except during the solar conjunction period lasting about a month) with data rates varying from 8 kbps to 32 kbps as the Earth–Venus range varies.

Three filters of the IR1 camera (Iwagami et al. 2011) with band passes centered at 0.9, 0.97 and 1.07 µm are used for surface imaging on the nightside in the CO_2_ windows where the atmosphere and the clouds are more transparent. The filters allow for estimating the near-surface abundance of water vapor using differential absorption at 0.97 and 1.01 µm (and limited by the large atmospheric opacity) as well as the spatial variation of surface emissivity using all three channels. Of the four filters of the IR2 cameras centered at 1.735, 2.02, 2.26 and 2.32 µm, the 1.735- and 2.26-µm filters together enable investigation of aerosol properties and the 2.26- and 2.32-µm filters are useful for CO abundance on the nightside. The 2.02-µm filter allows studying cloud-top elevation due to CO_2_ absorption on the dayside. The two filters of UVI allow a study of SO_2_ abundance above the clouds due to its absorption at 283 nm but not at 365 nm. However, there may be other absorbers which may also absorb differentially at these two wavelengths. All of these filters are useful to compare global contrast patterns or cloud morphology and measure cloud motions for improved understanding of the global atmospheric circulation at slightly different altitudes.

## Introduction

The cloud cover on Venus is ubiquitous, unlike that found on Earth and Mars. That Venus shows little contrast at most reflected wavelengths except ultraviolet (UV) has been known for a long time (Coffeen et al. 1971). The dayside contrast patterns observed are known to vary on short timescales. The dayside contrasts are wavelength dependent (200–600 nm, peaking at 365 nm and vanishing at the extremes of this range). Further, the contrasts vary with phase angle and are found to also be scale dependent. At shorter wavelengths (< 600 nm), contrast in global images peaks between 40° and 60° phase angles and at around 140° at longer wavelengths (> 550 nm) as measured from the unpolarized light images (Limaye 1984) from the Pioneer Venus Orbiter (PVO) Cloud Photopolarimeter (OCPP, Travis et al. 1979). The contrasts on Venus were noticed more than a century ago and recorded as drawings as early as 1881 (Niesten and Stuyvaert 1904). Venus was photographed by several observers in blue–violet as early as the 1920s (Ross 1927, 1928; Slipher 1931). Subsequently, when more frequent ultraviolet images were obtained, it was realized that Venus showed a lot of variability, and hence, the images revealed clouds rather than features on the surface (Claydes 1909). A brief review of cloud patterns in telescopic images of Venus has been presented by Dollfus (1975).

We present here the first views of Venus from the Akatsuki data that reveal this variability not only on the dayside, but also on the nightside from imaging at different wavelengths. The day- and nightside pattern differences stem from how they are observed. On the dayside clouds are imaged in reflected light and thus reveal patterns near the cloud tops, while the nightside clouds are observed in radiation emitted by the atmosphere below the clouds toward the surface. Thus, the nightside images reveal features caused by differences in opacity of the upper portions of the cloud layer and refer to the mid- and lower levels illuminated by radiation from the lower levels of the atmosphere at 1.74, 2.26 and 2.32 µm. Thus, nightside features may have different origins and are likely related to differences in cloud properties arising from trace species, cloud chemistry and microphysics and dynamics, somewhat different from cloud particle property variations in Earth clouds, which are primarily due to phase change related to vertical motions. At 0.9, 0.97 and 1.01 µm, the Venus atmosphere and clouds are somewhat transparent, and thus, radiation from the surface is detected in the nightside images.

### Dayside images of Venus

By now, global views of Venus have been obtained by many spacecraft missions at a limited number of wavelengths. Images at 365 nm were obtained from Mariner 10, the Pioneer Venus Orbiter, and Venus Express prior to Akatsuki. The first spacecraft images were obtained over an eight-day period during the Mariner 10 flyby of Venus in February 1974 (Murray et al. 1974). The morphology of the cloud cover from these images and their relationship to the Y features seen prominently in Earth-based images has been presented by Dollfus (1975). Most of the features identified in the Mariner 10 images during its short coverage were also seen in the longer duration of the Pioneer Venus Orbiter, and the morphology was presented by Rossow et al. (1980). PVO also obtained 25–40 km per pixel images (365-nm global images with a few limb images at 365 and 690 nm) and polarization data (~ 100 km per pixel) in reflected sunlight at four wavelengths (270, 365, 550 and 935 nm) from a spinning spacecraft and using the orbital motion of the spacecraft (Travis et al. 1979). The Venera 9 and 10 orbiters also obtained 17 images covering a limited portion of the Sun-lit planet during October 26, 1975–December 25, 1975, using the motion of the orbiters with a 512 element linear photometer array with 30° wide field of view at the violet and ultraviolet wavelengths (Keldysh 1977). The polarization mode intensity images showed morphology of the cloud cover to be similar at 270 and 365 nm in unpolarized light, but also showed features in polarization data at the two longer wavelengths (Limaye 1984) which were similar to those seen in the intensity images.

The orbiter infrared radiometer (OIR), a six-filter instrument on PVO, also yielded thermal infrared views of the northern hemisphere cloud tops at 10.6–12.6 µm for about 2 months (Taylor et al. 1980; Apt et al. 1980), but little small-scale detail was seen in the images constructed from these data. Later the Galileo orbiter took 77 images of Venus in reflected light from the solid-state imaging (SSI) camera (Belton et al. 1991) during its Venus gravity-assisted flyby over a very short period (2.5 days) in February 1990 at two different wavelengths in reflected light (418 and 986 nm effective wavelengths). The SSI images at 418 nm showed morphologies to be similar to the Mariner 10 (Murray et al. 1974) and Pioneer Venus observations (Rossow et al. 1980); however, at 986 nm some differences in morphology were seen (north–south linear patterns straddling the equator). The Venus Monitoring Camera (VMC, Markiewicz 2007) on the Venus Express Orbiter (Svedhem et al. 2007) imaged Venus at 365, 513, 950 and 1010 nm, and the cloud morphology has been presented by Titov et al. (2008, 2012). Dayside images of limited portions of Venus were also obtained by the VIRTIS instrument between 280 and 1000 nm with 6 nm spacing, but the internal scattering within the instrument impacted the data by reducing contrasts. Prior to Akatsuki’s arrival at Venus, the MESSENGER spacecraft also imaged Venus from its wide- and narrow-angle cameras in June 2007, providing more than two hundred images of Venus from the wide-angle camera at twelve wavelengths over about 2 days. Unfortunately the VMC instrument could not acquire good “in-orbit” additional flat fields to remove the image defects (Titov et al. 2012) during this period, so a comparison of VMC and MESSENGER concurrent images is not useful.

### Nightside images of Venus in near infrared

**Fig. 1:**
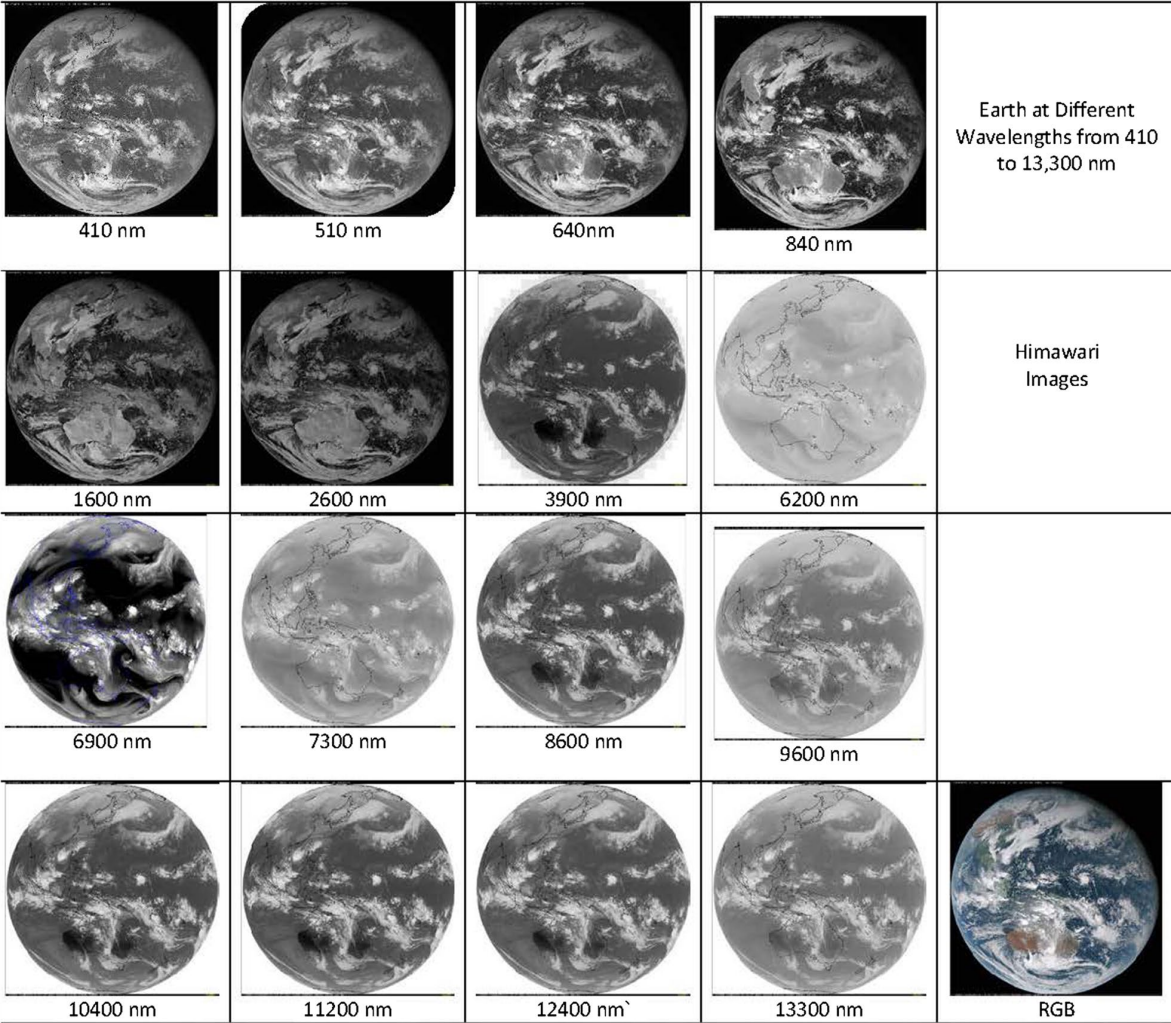
An example of Earth clouds imaged by the Himawari weather satellite (December 27, 2016, 04:00 UT) in geosynchronous orbit at sixteen different wavelengths. The cloud features show a high degree of similarity from 410 nm to 13.3 µm

**Fig. 2:**
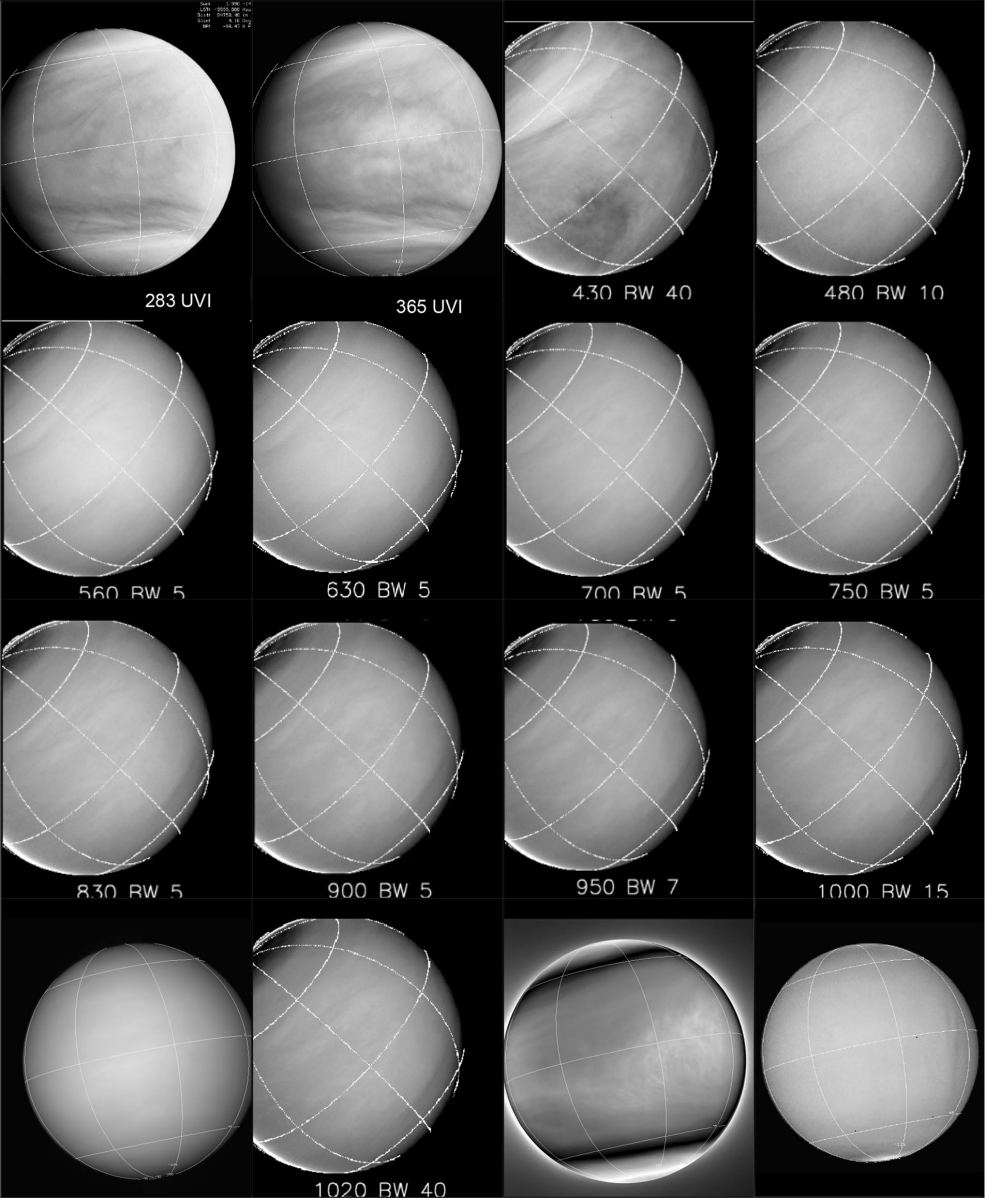
Venus at different wavelengths imaged by Akatsuki and MESSENGER cameras. Top row—(left to right) 283 nm (20160425_171339), 365 nm (20160425_171716) from the UVI camera on Akatsuki, and 430 nm and 480 nm (MESSENGER–MDIS/WA). Second row—560, 630, 700 and 750 nm (MDIS-WA). Third row—830, 900, 950 and 1000 nm (MDIS-WA). Bottom row—900 nm from Akatsuki (ir1_20160506_180208_09d), 1020 nm (MDIS-WA), 2020 nm from Akatsuki (ir2_20160506_180824_202) and 10–14 µm from Akatsuki (lir_201506_182053_pic). All MESSENGER images have been high-pass filtered to bring out the very low-contrast features. The MESSENGER images have been normalized using the Minnaert scattering function to bring out global-scale contrasts

First nightside images at near-infrared wavelengths of Venus showed surprising structures in Earth-based telescope observations (Allen and Crawford 1984). Galileo was the first spacecraft to map Venus between 0.7 and 5.2 µm from the near-infrared mapping spectrometer (NIMS) using the spacecraft spin and motion (Carlson et al. 1991). The mapping channel of the visible infrared thermal imaging spectrometer (VIRTIS) also on Venus Express imaged the planet between 0.28 and 5.0 µm during April 15, 2006–November 27, 2014 (Piccioni et al. 2007a), and provided the widest spectral coverage of the southern polar hemisphere of Venus at low spatial resolution and limited coverage of the low southern and equatorial northern latitudes at higher spatial resolution (as high as a few hundred meters at periapsis). Both VMC and VIRTIS predominantly provided a polar view of the Venus cloud cover at ~ 25–45 km per pixel size and more detailed views of the equatorial and northern latitudes at a spatial scale of as high as 1–2 km per pixel. The near-infrared data from VIRTIS showed details of the polar regions of Venus not readily accessible to Akatsuki. On the nightside of Venus, VIRTIS data revealed cloud contrasts in thermal emission from the cloud layer (Peralta et al. 2017) and also revealed cloud layer opacity variations above the lower clouds which are back-illuminated by emitted radiation from the warmer atmosphere below the cloud layer and the surface (Hueso et al. 2012). VIRTIS also revealed the intricacies of the inner region of the (southern) hemispheric vortex circulation (Garate-Lopez et al. 2015; Luz et al. 2011) which supplement the reflected light views that revealed the vortex from space–time composites of Mariner 10 dayside images (Suomi and Limaye 1978) and Galileo (Peralta et al. 2007). It is useful to note that on Earth, cloud morphologies look very similar from the blue to thermal infrared wavelengths (Fig. 1), unlike on Venus (Fig. 2). Images taken from the Himawari satellite at sixteen different wavelengths between 410 nm and 15 µm are shown in Fig. 1, while similar wavelength coverage for Venus is shown in Fig. 2 from recent missions. Unlike the Earth images which are concurrent, we have only limited concurrent coverage over the same spectral range from the MESSENGER flyby data which are supplemented by Galileo and Akatsuki for illustrative purposes. The MESSENGER images are taken from the MDIS wide-angle camera (Hawkins et al. 2009; Robinson et al. 2007) with narrow-band filters (10 nm), comparable to the band pass of the Akatsuki images at 283 and 365 nm (Table 1).

**Table 1:** Characteristics of cameras on Akatsuki orbiter

Camera	Channel name	Band center (μm)	Bandwidth (micron)	Transmittance	Pixel size (mm)	# Lines	# Samples	Focal length (mm)	Day/night
IR1	090d	0.900	0.00910	0.0027	0.017	1024	1024	84.2	Day
	090n	0.898	0.02890	0.74	0.017	1024	1024	84.2	Night
	097	0.969	0.03860	0.78	0.017	1024	1024	84.2	Night
	101	1.009	0.03910	0.75	0.017	1024	1024	84.2	Night
IR2	174	1.735	0.041	0.85	0.017	1024	1024	85.41	Night
	226	2.26	0.052	0.67	0.017	1024	1024	85.44	Night
	232	2.32	0.036	0.67	0.017	1024	1024	85.41	Night
	202	2.02	0.039	0.06	0.017	1024	1024	85.50	Day
	165	1.65	0.283	0.93	0.034	520	520	85.35	–
UVI	283	0.283	0.014	0.280	0.013	1024	1024	63.3	Day
	365	0.365	0.014	0.509	0.013	1024	1024	63.3	Day
LIR		10.00	4.00	–	0.037	328	248	42.2	Day and night

The difference between the wavelength similarity in the appearance of Earth and for Venus is striking. The differences are not due to the vertical extent of the clouds but due to different origins and chemical composition of the clouds on Earth and Venus. Venus clouds are complex as we have discovered. The ultraviolet contrasts were first discovered more than a century ago (Ross 1928), and the suggestion that the clouds were made of droplets containing sulfuric acid solutions (Young 1973) was confirmed more than 4 decades ago (Hansen and Hovenier 1974). But, we still do not know the species which are responsible for the dayside contrasts seen in the dayside cloud cover, except that sulfur dioxide gas is one of them and there is at least one other absorber which is as yet unknown (Travis 1975). Morphologies provide a good diagnostic tool for relating the atmospheric processes involved with the sources and sinks of the aerosols, condensates and trace gases. Chemical models developed for the Venus atmosphere include more than forty species and involve more than a hundred reactions (Krasnopolsky 2007; Mills and Allen 2007; Mills et al. 2007) which are dependent on ambient conditions, further complicating analysis and interpretation in light of incomplete data and information. Venera and VeGa landers, four Pioneer Venus probes and two VeGa balloons have sampled the clouds below 62 km and obtained useful measurements, but these are proving to be insufficient for understanding the Venus clouds.

The Akatsuki camera observations are providing high-quality global images of Venus at multiple wavelengths from an equatorial perspective of both north and south hemispheres, enabling a study of the global morphologies and shedding new light on the clouds of Venus. The Akatsuki orbiter’s cameras are now imaging Venus at thirteen wavelengths from 283 nm to 10 µm. These images provide global views of the day- and nightside of Venus at spatial scales from ~ 70 km to as high as 0.2 km per pixel. Concerning global cloud morphology, Akatsuki provides the best global views of Venus from a near-equatorial perspective at several wavelengths to date—283, 365, 900, 2020 nm on the dayside and at 0.97, 1.01, 1.74, 2.26 and 2.32 µm on the nightside, while the LIR camera provides day- and nightside views of Venus at the thermal wavelengths.

The altitudes of the features seen in day- and nightside images are not known with certainty. From analysis of PVO polarization data, the aerosol unit optical depth is at about 28 mb or at about 71 km in equatorial regions and at about 40 mb (~ 68 km) in polar regions (Kawabata et al. 1980). From the analysis of 1.6-µm VIRTIS data, Ignatiev et al. (2009) found the cloud tops located at 74 ± 1 km in low and midlatitudes and at 63–69 km poleward of ± 50° latitude. Tracking clouds and matching their zonal speeds to entry probe zonal wind from Pioneer Venus probes or cyclostrophic wind from thermal structure data have often been invoked to estimate the vertical level of the features (Belton et al. 1991; Hueso et al. 2015; Limaye 1985). Using a cloud model, Takagi and Iwagami (2011) have explored the causes for contrasts in the images acquired by IR1 and IR2 Akatsuki cameras in near infrared at 0.9 and 2.02 µm on the dayside and at 2.26 µm on the nightside. They concluded that the dayside 0.9 µm contrasts are due to variations of the cloud optical thickness as is also the case for the nightside 2.26 µm contrasts. They suggest that 2.02 µm contrasts depend on not only the cloud altitude, but also the cloud optical thickness. They also suggest that although Belton et al. (1991) inferred that dayside 0.986 µm contrasts correspond to about 55 km using the vertical wind shear, the contrasts could be located anywhere in the cloud from 48 to 70 km. Similarly, although Carlson et al. (1991) attribute the 2.3 µm contrasts to 50 km, Takagi and Iwagami (2011) suggest that the contrasts may be located anywhere in the mid- or upper cloud region. Hueso et al. (2015) attribute the cloud patterns between 0.3 and 1 µm to altitudes between 60 and 72 km and their near-infrared cloud motion measurements match those from the Pioneer Venus probe and VeGa balloon derived winds at altitudes between 56 and 62 km, differing from the 58–64 km level derived by (Sánchez-Lavega et al. 2008) from radiative calculations. It is quite possible that these discrepancies are due to temporal and local time variations or from the global variations in the cloud structure (as indicated by the Akatsuki nightside images) that can be at odds with cloud models deployed to infer the altitudes.

The morphological information at multiple wavelengths enabled by the Akatsuki cameras presented here provides new constraints on the evolution and origins of the contrasts—both absorbers of incident solar radiation responsible for dayside patterns and local variations in cloud opacity and thermal contrasts responsible for the nightside patterns. We describe below the global morphology of the dayside cloud cover of the cloud-top region and present the nighttime morphology of the cloud features in the mid- and lower levels of the cloud cover.

## Akatsuki multispectral images of Venus: cameras onboard

There are five cameras on Akatsuki to image Venus at the ultraviolet to thermal infrared wavelengths—UVI [Yamazaki et al., submitted to EPS, Akatsuki special issue], IR1 (Iwagami et al. 2018, submitted to EPS Akatsuki special issue), IR2 (Satoh et al. 2016), LIR (Fukuhara et al. 2011, 2017b) and the Lightning and Airglow Camera (LAC) which does not produce an image (Takahashi et al. 2008), and is used when the nightside of Venus is visible to Akatsuki to detect lightening and airglow. Table 1 provides the characteristics of the four imaging cameras. The 1.65-µm filter in the IR2 camera was used for zodiacal imaging during cruise and is not used to image Venus and hence is listed in this table for completeness only. The focal lengths are nominal values for IR1, UVI and LIR cameras and have been updated from star fields during the mission for the IR2 camera. Updates are expected for the other cameras. During the long interval between the first attempt in December 2010 and the successful orbital insertion in December 2015, Akatsuki came much closer to the Sun than anticipated with 11 perihelion passages. Thus, the camera performance was somewhat affected, particularly for the IR1 camera, requiring new procedures for data calibration.

Generally, when the Akatsuki orbiter predominantly views the night hemisphere of Venus from its position in its orbit, the UVI camera is not used, and only the IR1 and IR2 cameras image the nightside of Venus. The two 0.9-µm filters (09d and 09n) on the IR1 camera have different band passes (Table 1) to enable imaging of the day- and nightsides due to the differences in the reflected and emitted radiation. The LIR camera images Venus throughout the orbit—day and night—as it senses radiation emitted to space by the planet from the cloud tops. Dayside images are taken at 283 and 365 nm (UVI) wavelengths as well as at 0.9 µm (IR1) and 2.02 µm (IR2). Instrumental constraints prevent concurrent images from different filters from the same camera, but the time interval is relatively short (about 214 s for UVI) which provides near simultaneous imaging at different wavelengths except at close approach to Venus. The performance of the four cameras during the first year of operations in orbit is presented in this special issue by Iwagami et al. (IR1), Satoh et al. (2017) for the IR2, Yamazaki et al. (UVI) and Fukuhara et al. (2017b) for the LIR camera. An electronics problem with a unit in late 2016 has affected the operation of the IR1 and IR2 cameras, and while efforts continue to address this problem, the cameras have not sent back any data since the anomaly.

## Temporal coverage

**Fig. 3:**
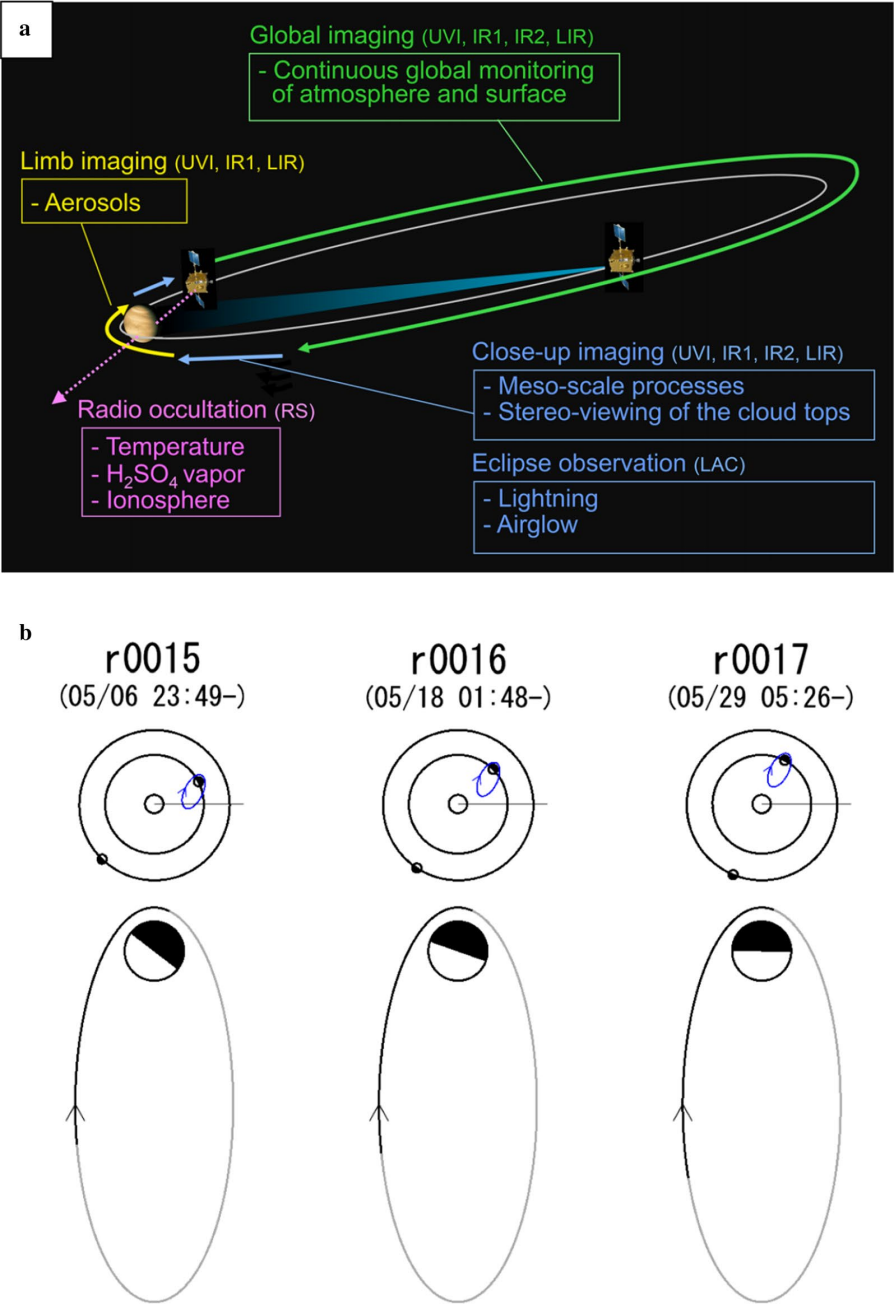
**a** Schematic view of orbital path of the Akatsuki orbiter around Venus and general observing plan during the ~ 10.5-day orbit. **b** A plan view of the orbits of Earth (outer ellipse), Venus (inner ellipse) and Akatsuki for orbit numbers 15, 16 and 17. An expanded view of three consecutive Akatsuki orbits around Venus is shown with the arrow pointing in the direction of the spacecraft in orbit. The dates and times indicate periapsis times. The dark shading in the circle represents the Venus nightside. Note that the size of Venus is exaggerated as compared to the size of the Akatsuki orbit for clarity. Similarly, in the upper panel the size of the Akatsuki orbit is also not to scale compared to the Earth and Venus orbits. The darker portion of the orbit indicates times when the orbiter is above the Venus equatorial plane. Sun avoidance zone for the high-gain antenna and the radiative cooler for the IR2 camera pose constraints on the pointing of the spacecraft when viewing Venus

Four hours after the Venus orbit insertion (VOI), the IR1, UVI and LIR cameras took Venus images. Only IR2 needed to be cooled down for 10 days and took the “first light” images after a long hibernation period in January 2016. Systematic data collection began on April 1, 2016, from its near-equatorial orbit with a periapsis altitude of ~ 1000 km and a 10.5-day period and an eccentricity of about 0.92 (Fig. 3a). A schematic diagram of the Akatsuki orbit around Venus is shown in Fig. 3b for three of its orbits to illustrate when Akatsuki observes the day or the nightside of the planet. Routine imaging from all four cameras has been performed on every orbit except during solar conjunctions when communications are interrupted between Akatsuki and the Usuda DSN. One advantage of the longer orbit is that it is possible to monitor the cloud patterns with fewer gaps as Akatsuki observes Venus’ day and night hemispheres continuously from similar vantage points for longer duration as compared to the 24-h eccentric polar orbit of Venus Express.

In November 2016, a technical problem prevented imaging from the IR1 and IR2 cameras which share some electronics, and currently, those two cameras are not returning any data. The cadence of most of the images from the UVI, IR1 and IR2 cameras is 2 h, but some images have been acquired 1 h apart. Generally, the UVI camera makes three exposures for one “image” which takes 210 s and a median filter is applied onboard to reduce the dark noise. At close approach to Venus, the median filter step is skipped and images can be acquired more quickly (150 s apart). The LIR camera can image Venus far more rapidly than other cameras—every 5 s. Onboard storage constraints, data communication rates and instrument operations limit the sampling interval and total number of images from the UVI, IR1 and IR2 cameras. Different data acquisition modes have been developed to account for the data communication rates due to Earth–Venus range. The LIR camera takes more frequent images, but the data volume is smaller due to the smaller size of its detector. The total number of images returned from Akatsuki is thus somewhat less than the number of images returned from VMC and provides global views of the planet as compared to the mostly near-polar perspective views imaged near the apoapsis portion of the Venus Express orbit when the day and night hemispheres were contained in a single VMC frame.

Table 2 provides a list of the images used in this paper along with some geometry information—range of the Akatsuki orbiter from Venus, the spacecraft sub-point latitude and longitude, the sub-solar latitude and longitude, the azimuth angle of the Venus north pole and the pixel size at the sub-spacecraft location (km).

**Table 2:** Geometry information for the Venus images

Figure #	ID	IMAGE	Orbit	Range (km)	SubLat	SubLon	SunLat	SunLon	NP AZM	Venus size (°)	PixSiz (km)
2	a	uvi_20160425_171339_283	13	84758.4	2.9	235.8	2.0	213.9	− 98.5	4.2	17.4
	b	uvi_20160425_191715_365	13	66,816.1	4.1	230.3	2.0	214.2	− 98.2	5.3	13.7
4	a	uvi_20160425_171339_283	13	84,758.4	2.9	235.8	2.0	213.9	− 98.5	4.2	17.4
	b	uvi_20160425_171716_365	13	84,264.8	2.9	235.7	2.0	213.9	− 98.5	4.2	17.3
	c	uvi_20160506_181341_283	14	69,448.1	4.7	240.6	1.4	247.8	− 101.1	5.1	14.3
	d	uvi_20160506_181716_365	14	68,919.6	4.8	240.4	1.4	247.8	− 101.1	5.1	14.2
	e	uvi_20161120_132347_283	32	68,174.9	12.2	178.9	2.5	138.1	− 95.0	5.2	14.0
	f	uvi_20161120_132721_365	32	67,616.8	12.2	178.8	2.5	138.1	− 95.0	5.2	13.9
5	a	uvi_20161223_101110_283	35	71,763.7	12.9	210.8	1.0	239.1	69.1	4.9	14.7
	b	uvi_20161223_101445_365	35	71,263.3	13.0	210.6	1.0	239.1	69.1	5.0	14.6
	c	uvi_20170104_060111_283	37	101,196.7	− 13.2	318.7	0.1	275.5	84.1	3.5	20.8
	d	uvi_20170104_060444_365	37	101,603.2	− 13.1	318.6	0.1	275.5	84.1	3.5	20.9
	e	uvi_20170114_170111_283	37	74,801.2	13.8	241.0	− 0.6	307.9	76.8	4.7	15.4
	f	uvi_20170114_170446_365	37	74,313.7	13.9	240.7	− 0.6	307.9	76.8	4.7	15.3
6	a	uvi_20170115_083111_283	38	93,172.9	− 13.6	334.6	− 0.7	309.8	83.2	3.8	19.1
	b	uvi_20170115_083445_365	38	93,604.5	− 13.6	334.5	− 0.7	309.9	83.2	3.8	19.2
	c	uvi_20170121_103110_283	38	368,626.1	0.3	297.5	− 1.1	328.7	89.8	1.0	75.7
	d	uvi_20170121_103443_365	38	368,592.1	0.3	297.5	− 1.1	328.7	89.8	1.0	75.7
	e	uvi_20161223_141058_283	35	33,943.9	17.8	177.1	1.0	239.6	80.4	10.4	7.0
	f	uvi_20161223_141334_365	35	33,484.6	17.9	176.3	1.0	239.6	80.4	10.6	6.9
7	a	ir1_20160425_170207_09d	13	86,321.4	2.8	236.2	2.0	213.9	− 98.5	4.1	17.4
	c	ir1_20160506_180208_09d	14	71,138.0	4.6	241.3	1.4	247.8	− 101.2	5.0	14.4
	e	ir1_20160517_200207_09d	15	68,970.5	5.9	250.1	0.6	282.0	− 97.4	5.1	13.9
8	a	ir2_20160506_180824_202	14	70,223.6	4.7	241.0	1.4	247.8	− 101.9	5.0	14.2
	c	ir2_20160517_080822_202	15	148,368.0	1.7	271.4	0.7	280.4	− 99.8	2.4	30.0
	e	ir2_20160621_220821_202	19	228,037.7	− 7.5	6.3	− 1.8	30.5	− 86.8	1.5	46.0
9	a	ir1_20160916_012708_09d	27	18,272.0	− 3.6	273.5	0.1	297.2	− 74.2	19.7	3.7
	b	ir1_20161019_054807_09d	29	10,716.3	10.1	5.1	2.2	39.1	− 73.7	35.0	2.2
	c	uvi_20160927_044558_365	28	16,466.9	− 1.0	300.1	0.9	331.5	− 73.5	21.9	3.4
	d	uvi_20160927_045753_365	28	17,158.5	− 5.1	286.0	0.9	331.5	− 75.5	21.0	3.5
	e	ir1_20160506_220208_09d	14	30,485.4	9.6	209.2	1.4	248.3	− 97.1	11.6	6.2
	f	ir1_20160303_212359_09d	9	11,631.3	2.3	358.6	1.9	51.9	− 82.8	31.9	2.3
10	a	ir2_20160506_220822_202	14	29,238.3	9.9	207.0	1.4	248.3	− 97.8	12.1	5.9
	c	ir2_20160824_203242_202	25	22,522.3	− 15.0	185.8	− 1.5	228.8	− 81.0	15.8	4.5
	e	ir2_20161008_062241_202	28	13,552.0	6.1	339.1	1.6	5.4	− 65.3	27.0	2.7
	Row, Col										
11	1,1	uvi_20160425_171339_283	13	84,758.4	2.9	235.8	2.0	213.9	− 98.5	4.2	17.4
	1,2	uvi_20160425_171339_283	13	84,758.4	2.9	235.8	2.0	213.9	− 98.5	4.2	17.4
	1,3	ir1_20160425_170207_09d	13	86,321.4	2.8	236.2	2.0	213.9	− 98.5	4.1	17.4
	1,4	ir2_20160425_170821_202	13	85,478.7	2.8	236.0	2.0	213.9	− 99.2	4.1	17.3
	2,1	uvi_20160506_181341_283	14	69,448.1	4.7	240.6	1.4	247.8	− 101.1	5.1	14.3
	2,2	uvi_20160506_181716_365	14	68,919.6	4.8	240.4	1.4	247.8	− 101.1	5.1	14.2
	2,3	ir1_20160506_180208_09d	14	71,138.0	4.6	241.3	1.4	247.8	− 101.2	5.0	14.4
	2,4	ir2_20160506_180824_202	14	70,223.6	4.7	241.0	1.4	247.8	− 101.9	5.0	14.2
	3,1	uvi_20160517_201339_283	15	67,313.8	6.1	249.3	0.6	282.0	− 97.3	5.2	13.8
	3,2	uvi_20160517_201715_365	15	66,792.8	6.1	249.0	0.6	282.0	− 97.3	5.3	13.7
	3,3	ir1_20160517_200207_09d	15	68,970.5	5.9	250.1	0.6	282.0	− 97.4	5.1	13.9
	3,4	ir2_20160517_200822_202	15	68,075.0	6.0	249.6	0.6	282.0	− 98.1	5.2	13.7
		uvi_20160506_221340_283	14	28,171.4	10.1	205.0	1.4	248.4	− 97.0	12.6	5.8
12		uvi_20160506_221716_365	14	27,443.6	10.2	203.6	1.4	248.4	− 97.1	12.9	5.6
		ir2_20160506_220823_202	14	29,235.0	9.9	207.0	1.4	248.3	− 97.8	12.1	5.9
13	a	ir2_20160325_073211_174	11	109,374.9	− 9.1	246.0	2.6	117.6	− 91.1	3.2	22.1
	b	ir2_20160325_073333_226	11	109,535.9	− 9.1	246.0	2.6	117.6	− 91.1	3.2	22.1
	c	ir2_20160325_074039_232	11	110,369.4	− 9.1	245.9	2.6	117.6	− 91.1	3.2	22.3
	d	ir2_20160507_040211_174	15	56,594.8	− 11.2	339.5	1.4	249.1	− 93.9	6.2	11.4
	e	ir2_20160507_040333_226	15	56,816.2	− 11.2	339.4	1.4	249.1	− 93.9	6.2	11.5
	f	ir2_20160507_041036_232	15	57,952.3	− 11.2	338.7	1.4	249.1	− 93.9	6.1	11.7
	g	ir2_20160712_020212_174	20	71,695.2	8.0	347.1	− 2.6	93.1	− 91.8	4.9	14.5
	h	ir2_20160712_020333_226	20	71,489.0	8.1	347.0	− 2.6	93.1	− 91.8	4.9	14.4
	i	ir2_20160712_021127_232	20	70,275.8	8.2	346.7	− 2.6	93.1	− 91.8	5.0	14.2
	j	ir2_20160904_170212_174	25	57,016.7	11.5	42.3	− 0.7	262.3	− 105.1	6.2	11.5
	k	ir2_20160904_180128_226	25	47,590.3	12.7	34.8	− 0.7	262.4	− 104.0	7.4	9.6
	l	ir2_20160904_171123_232	25	55,595.2	11.6	41.3	− 0.7	262.3	− 105.1	6.4	11.2
14	a	ir2_20160905_033211_174	26	70,896.3	− 13.9	153.7	− 0.7	263.6	− 93.3	5.0	14.3
	b	ir2_20160905_033333_226	26	71,087.9	− 13.9	153.6	− 0.7	263.7	− 93.3	5.0	14.4
	c	ir2_20160905_034120_232	26	72,174.1	− 13.8	153.1	− 0.7	263.7	− 93.3	4.9	14.6
	d	ir2_20160927_090209_174	28	56,626.7	− 15.6	191.8	0.9	332.0	− 95.7	6.2	11.4
	e	ir2_20160927_090331_226	28	56,837.6	− 15.6	191.6	0.9	332.0	− 95.7	6.2	11.5
	f	ir2_20160927_091120_232	28	58,038.0	− 15.5	190.8	0.9	332.0	− 95.7	6.1	11.7
	g	ir2_20161008_090056_174	29	38,632.6	− 18.1	215.7	1.6	5.7	− 108.3	9.2	7.8
	h	ir2_20161008_090127_226	29	38,728.2	− 18.1	215.6	1.6	5.8	− 108.3	9.1	7.8
	i	IRTF Cont_K April 27	–	61387346.	− 3.1	26.0	− 0.1	263.4	337.2	*20.6*	34.5
	j	ir2_20161030_080211_174	31	66,851.7	− 15.8	215.0	2.5	73.0	− 94.9	5.3	13.5
	k	ir2_20161030_080333_226	31	67,066.9	− 15.8	214.9	2.5	73.0	− 94.9	5.3	13.5
	l	IRTF Cont_K April 28	–	63417688.	− 2.8	29.8	0.1	269.6	337.2	*19.9*	35.7
15, 16		ir2_20160415_080211_174	13	66,540.4	− 10.8	289.1	2.4	182.0	− 92.1	5.3	13.4
		ir2_20160415_080333_226	13	66,757.9	− 10.7	289.0	2.4	182.0	− 92.1	5.3	13.5
		ir2_20160415_081039_232	13	67,881.7	− 10.7	288.7	2.4	182.0	− 92.1	5.2	13.7
17		ir2_20161019_143211_174	30	95,667.9	− 13.2	195.3	2.2	40.2	− 99.0	3.7	19.3
		ir2_20161019_143332_226	30	95,837.9	− 13.1	195.2	2.2	40.2	− 99.0	3.7	19.3
		ir2_20161019_144120_232	30	96,816.5	− 13.1	195.0	2.2	40.2	− 99.0	3.6	19.5
18		ir2_20160927_110209_174	28	74,082.1	− 14.3	181.8	0.9	332.3	− 98.3	4.8	15.0
		ir2_20160927_110331_226	28	74,269.7	− 14.3	181.7	0.9	332.3	− 98.3	4.7	15.0
		ir2_20160927_111119_232	28	75,335.6	− 14.2	181.2	0.9	332.3	− 98.3	4.7	15.2
19	a	lir_20160723_073128_pic	22	63,970.5	− 14.3	70.6	− 2.6	128.0	− 83.4	5.5	56.1
	b	lir_20160506_182053_pic	14	68,384.1	4.8	240.2	1.4	247.9	− 101.2	5.2	60.0
20	a	uvi_20160515_171715_365	15	289,143.0	− 1.7	285.7	0.8	275.4	− 99.5	1.2	59.4
		uvi_20160517_201715_365	15	66,792.8	6.1	249.0	0.6	282.0	− 97.3	5.3	13.7
	b	ir2_20160506_180824_202	14	70,223.6	4.7	241.0	1.4	247.8	− 101.9	5.0	14.2
	c	lir_20160926_231748_pic	27	65,802.1	12.1	77.6	0.9	330.7	− 102.0	5.4	13.3
		lir_20160825_001606_pic	27	61,534.8	− 14.4	137.1	− 1.5	229.3	− 90.9	5.7	12.4

## Dayside morphology

Unlike on Earth where the clouds seen in reflected light are visible because of light scattering, the contrast features on Venus are seen due to absorption and scattering. By cloud morphology, we refer here to the patterns of these contrast features. Below we present the global cloud-top morphology from dayside equatorial views at 283 and 365 nm (UVI) and at 0.9 (IR1) and 2.02 µm (IR2). Most of the global images were obtained at about 15–20 km/pixel scale (Table 2) at the sub-spacecraft point, while two are at much lower resolution (~ 75 km/pixel). As the contrast on finer spatial scales (~ 5–10 km) is markedly different, we also present some samples of higher-resolution images at all wavelengths to compare with some earlier observations. Venus was previously imaged at high spatial resolution from Mariner 10 and Venus Express VMC and VIRTIS, and now, Akatsuki also provides views at 0.9 and 2.02 µm with spatial scale of ~ 2 km per pixel.

### Images at 283, 365 nm (UVI)

**Fig. 4:**
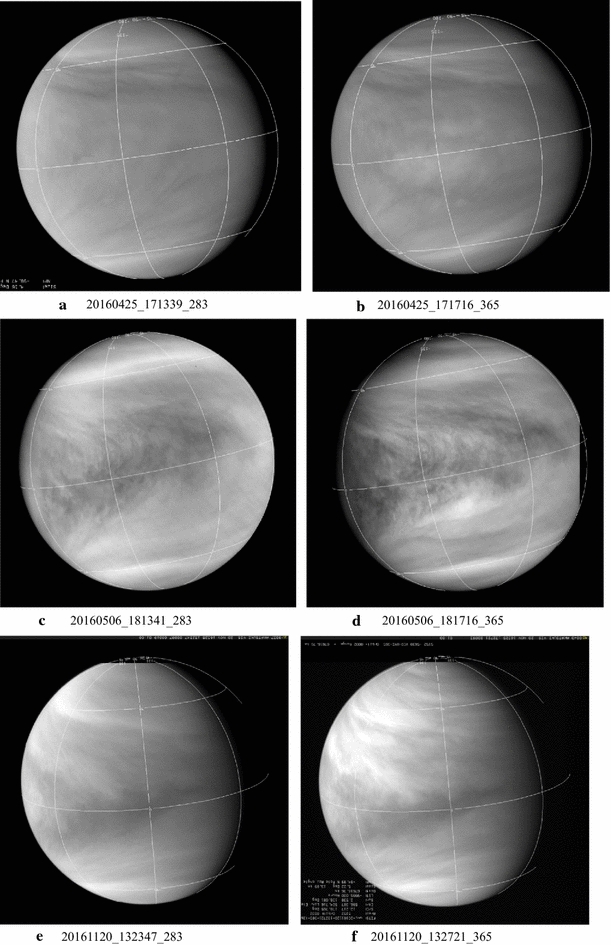
Selected images from the UVI camera taken from the 283 nm (left column) and 365 nm (right column). Latitude and longitude lines are shown with 45**°** spacing. (North pole is at the bottom.) Generally images taken through the 283- and 365-nm filters are very close together in time. Simultaneous imaging is not possible from the camera in the two filters which occupy different locations on the filter wheel in the camera and the time difference between them is about 150 s. At very close approach to Venus, complete overlap between 283- and 365-nm images is not possible due to the fast movement of Akatsuki it its orbit. All of the images are shown in calibrated versions with latitude–longitude overlays (north hemisphere is at the bottom in most images) and they have been scaled to the fit the frames. Geometry information is given in Table 2 for the displayed images

**Fig. 5:**
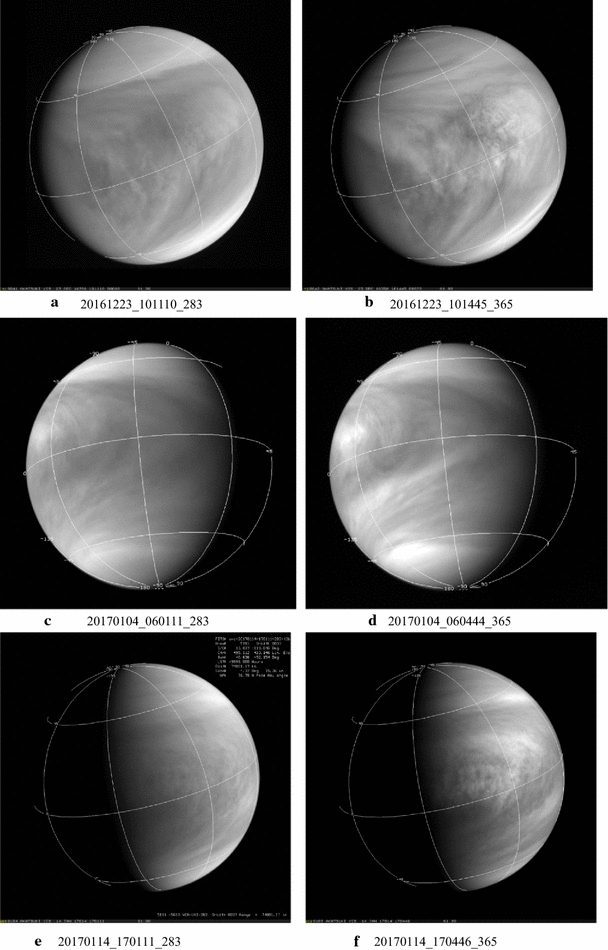
Selected images from the UVI camera taken from the 283 nm (left column) and 365 nm (right column). Latitude and longitude lines are shown with 45**°** spacing. (North pole is at the bottom.) Generally images taken through the 283- and 365-nm filters are very close together in time. Simultaneous imaging is not possible from the camera in the two filters which occupy different locations on the filter wheel in the camera and the time difference between them is about 150 s. At very close approach to Venus, complete overlap between 283- and 365-nm images is not possible due to the fast movement of Akatsuki it its orbit. All of the images are shown in calibrated versions with latitude–longitude overlays (north hemisphere is at the bottom in most images) and they have been scaled to the fit the frames. Geometry information is given in Table 2 for the displayed images

**Fig. 6:**
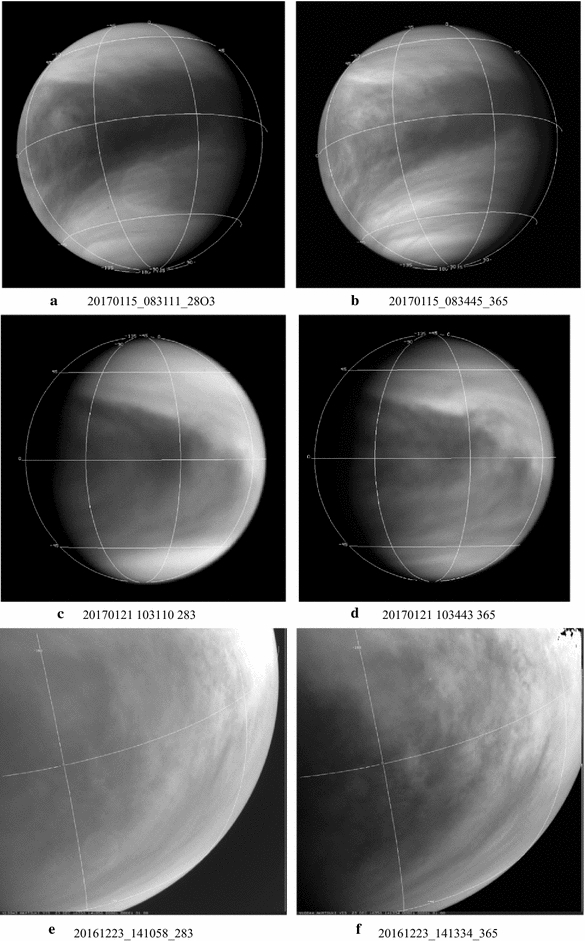
Selected images from the UVI camera taken from the 283 nm (left column) and 365 nm (right column). Latitude and longitude lines are shown with 45**°** spacing. (North pole is at the bottom.) Generally images taken through the 283- and 365-nm filters are very close together in time. Simultaneous imaging is not possible from the camera in the two filters which occupy different locations on the filter wheel in the camera and the time difference between them is about 150 s. At very close approach to Venus, complete overlap between 283- and 365-nm images is not possible due to the fast movement of Akatsuki it its orbit. All of the images are shown in calibrated versions with latitude–longitude overlays (north hemisphere is at the bottom in most images) and they have been scaled to the fit the frames. Geometry information is given in Table 2 for the displayed images

A representative sampling of 283- and 365-nm image pairs obtained at almost the same time is shown in Figs. 4, 5 and 6. Most of the images shown in Figs. 4, 5 and 6 were taken at low phase angles to show as much of the dayside hemisphere as possible and thus are not the highest resolution images from Akatsuki. Because of the circulation period of the atmosphere at the cloud-top level (~ 4 days) and the orbital period of Akatsuki (~ 10.5 days), full-frame global views of the dayside of Venus are intermittent and smaller images of Venus are obtained in between these occasions. For clarity, all of the global images are scaled to have the same size, but they differ in spatial resolution (see Table 2). The 365-nm images show the familiar contrast features, but continuous monitoring of Venus at 283 nm is new from Akatsuki. In the 283 images, Venus looks not too different from its appearance at 365 nm. Previously only low-resolution (~ 100–160 km per pixel) coverage at 270 nm was obtained in the polarimetry mode from Pioneer Venus (Kawabata et al. 1980) and a similar resemblance was also noted (Limaye 1984) between 270 and 365 nm maps. Generally, the images shown in Figs. 4, 5 and 6 show the “Y Feature” in its many incarnations over a period of about 9 months covered at both wavelengths. The Y feature is marked by bright arms inclined at a variable angle about the equator and often enclosing a region of a bright patch (e.g., images c, d, c, d, a, b) which may include bow-shaped features (e.g., a, b). The angle of the bright arms can be very high (e.g., c, d) and sometimes very low (e.g., e, f). The region between the two arms of the Y can be relatively narrow (a, b) or quite wide (c, d) and can have few contrast features (c, d) or many (a, b). The relative magnitude of the contrasts can also be different at the two wavelengths. Preliminary cloud motion measurements from the images at 283 and 365 nm reveal some differences, which may be due to altitude but could also be due to differences and changes in morphology (sources or processes producing the contrasts).

At the first impression, the cloud forms or contrasts seen at 283 and 365 nm are very similar; however, some differences in the relative brightness and contrasts are apparent (e.g., pairs a–d, k, l). Preliminary quantitative comparisons of colocated intensities at the two wavelengths reveal both positive and negative correlations that appear to vary with latitude. Such a study is currently underway. What is not yet known with confidence is the origins of the contrasts, i.e., what are the absorbers that cause these contrasts by selectively absorbing certain wavelengths in the incident solar radiation. It has been known that SO_2_ is expected to absorb some of the radiation below 330 nm, along with another absorber (Esposito 1980; Esposito et al. 1983; Travis 1975). Cloud-top level is believed to be similar at these wavelengths (estimated from images, but cannot be precisely determined due to the diffuse nature of the cloud-top region and the presence of haze), so the differences in morphology at the two wavelengths generally imply differences in distributions of opacity sources horizontally and vertically above the visible cloud tops. Variations in the abundance of SO_2_ above the cloud tops (90–110 km altitude) should affect the contrasts at 283 nm compared to 365 nm. SO_2_ abundance has been observed to vary rapidly above the cloud tops on a timescale of ~ an hour (Encrenaz et al. 2013, 2016; Marcq et al. 2011), so variation in contrasts at 283 nm on similar timescales can be expected. That we see comparable changes at 365 nm also indicates that other unknown absorbers besides SO_2_ are present and also contribute to the contrasts. Zhang et al. (2010) propose that sulfuric acid in the cloud droplets can undergo photolysis to produce sulfur oxides and loft them above the clouds tops. This can be another cause of rapid changes in the clouds.

More than a dozen absorbers have been suggested so far as candidates for the other unknown absorber(s) besides SO_2_ (Esposito et al. 1983), and while sulfur (Carlson 2016) and FeCl_3_ (Krasnopolsky 2016, 2017) have been favored as leading candidates for the ultraviolet absorption, there is no confirmation of their presence. There are other absorbers of incident sunlight at *λ* < 600 nm and some of these have been detected in the atmosphere of Venus (e.g., CS_2_). It is worth pointing out that although the literature refers to “ultraviolet” absorber(s), the absorption of incident solar radiation in the Venus atmosphere becomes noticeable at wavelengths shorter than 500 nm (Perez-Hoyos et al. 2013) or even 600 nm (Carlson 2016). Further, the nature of the other absorbers (besides SO_2_)—particulate, vapor or even microorganisms—is not known. Recently, a possibility of microorganisms contributing to absorption of sunlight in the clouds and also to contrasts has been raised by Limaye et al. (2017), based on the consideration that the known physical, chemical and spectral properties of the Venus cloud particles can be matched by terrestrial microorganisms that may have evolved on Venus when it had liquid water on its surface and migrated to the clouds. Earth clouds harbor many microorganisms and have been detected at altitudes as high as 41 km (Wainwright et al. 2003), so a similar existence in the clouds of Venus cannot be excluded.

In general, the contrast of the images is somewhat lower at 283 nm than at 365 nm. This is somewhat surprising considering that the intensity data acquired from the Pioneer Venus Orbiter Cloud Photopolarimeter showed somewhat larger contrasts at 270 nm compared to 365 nm, at least when contrast was defined in terms of absolute deviation from the intensity expected from a Minnaert law behavior (Limaye 1984). Despite the somewhat lower contrast, 283-nm images show generally the same morphology as 365-nm images, but the smaller-scale details are muted or absent. Visual examination of the images at these two wavelengths shows that a feature seen at one wavelength is absent in the other wavelength in certain regions at certain times. At other times or locations, the features appear to match well at the two wavelengths. Quantitative analysis of colocated 283- and 365-nm (near) simultaneous images shows the correlation of brightness values at the two wavelengths to be variable and part of the variation appears to be latitude dependent. A similar situation was found with Galileo and MESSENGER data at other wavelengths. The correlation between the wavelengths covered by Galileo and MESSENGER images was also found to vary. During the Galileo flyby there seemed to be an anticorrelation in brightness between the violet and near-infrared images (Belton et al. 1991), but this anticorrelation was not apparent during the MESSENGER flyby Peralta et al. (2017).

The Akatsuki images at 283 and 365 nm show many features previously observed—discrete smaller-scale features at low latitudes, bright spiraling streaks and bands at midlatitude, bow-like features near the sub-solar point and the Y-like feature in different shapes. As before, the contrasts are lower near the terminators. Lee et al. (2017) have conducted an analysis of the absorption and scattering properties of the clouds from Akatsuki images at 283 and 365 nm and further studies are being conducted.

### Dayside images at near-infrared wavelengths

The two near-infrared wavelengths, 0.9 and 2.02 µm wavelengths are somewhat less susceptible to scattering from the submicron haze that is found above the cloud tops and mixed in with the ~ 1.1-µm-radius particles of the cloud-top layer on Venus. Thus, any discrete features seen at these two wavelengths can be taken as an indication of the presence of somewhat larger particles which are also known to exist (Knollenberg et al. 1980) and inferred from VMC data (Petrova et al. 2015). Further, at 2.02 µm there is some absorption by CO_2_, so any darker patches seen in the images at these wavelengths may be indicative of some vertical relief in the cloud particle distribution.

#### 0.9 µm images (IR1)

**Fig. 7:**
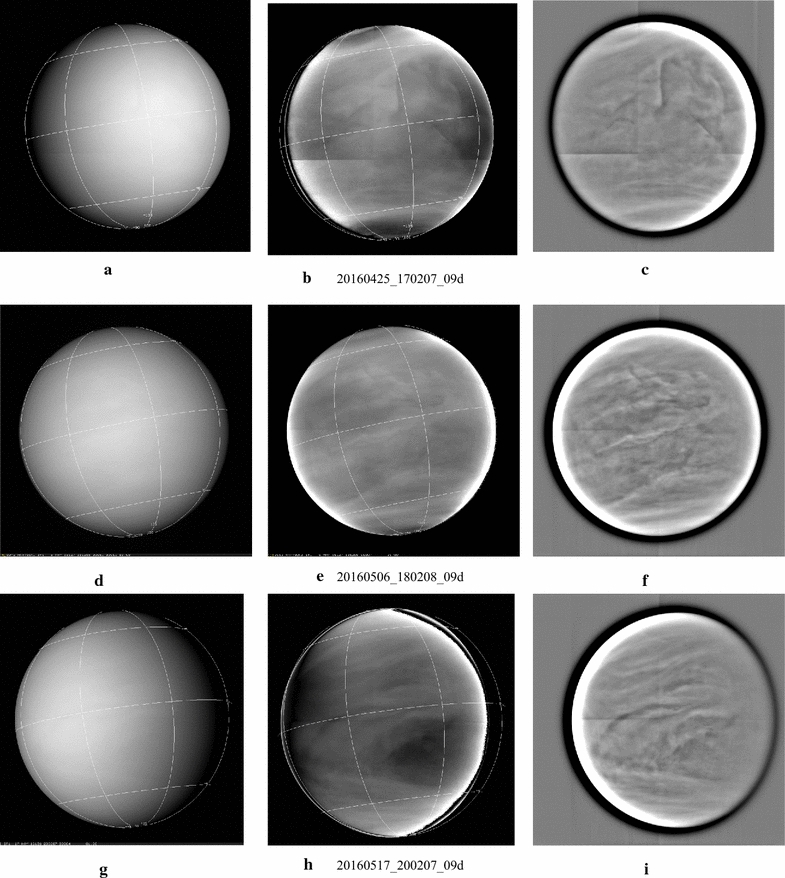
Selected global images taken at 0.97 µm on the dayside from the IR1 camera through the 09d filter. Calibrated images are shown in the left column and brightness normalized versions are shown in the middle column and high-pass filtered versions in the right column. Latitude and longitude lines are shown with 45**°** spacing. (North pole is at the bottom.) In the brightness normalized versions, the shading due to illumination and viewing angles has been removed using a Minnaert function. Local details become more visible in the high-pass filtered versions. In general, contrasts are much lower at 900 nm than at 365 nm as expected and the spatial scales and morphology are similar. However, the global organization is not as apparent as at 365 nm. In images taken closer to the planet which show Venus at ~ 2–6 km/pixel scale, very muted contrasts are seen. This is different from that observed at the ultraviolet wavelengths where the contrasts are much weaker on the very small spatial scales of ~ 5–10 km, and are seen only over scales that are about 10 times larger. Because Akatsuki spends only a short time at close proximity to Venus in its highly elliptic orbit, global coverage at high spatial resolution is not feasible as the camera’s field of view is too small as compared to the angular size of the planet as Akatsuki moves very quickly relative to the planet

Three representative global images taken through the 09d filter on the dayside of Venus are shown in Fig. 7. Original (calibrated) images are shown in the left column and show almost no detail, but contrast features can be seen in the brightness normalized versions which are shown in the middle column using the Minnaert law (Minnaert 1941). High-pass filtered versions are shown in the right column and show more local detail. Both the normalized and high-pass filtered versions reveal the linear boundaries of the four quadrants of the IR1 CCD due to the slight mismatch in the gains of the separate readout electronics for each quadrant. This mismatch was not revealed in the pre-launch ground calibration and appears to have developed during the long cruise and higher than planned solar exposure experienced by the Akatsuki orbiter during the first missed opportunity and the orbit insertion in December 2015 (Nakamura et al. 2016).

As expected from previous observations (Coffeen et al. 1971), the full-disk image of Venus shows little detail as the contrast is very low. Low to medium spatial resolution full-disk images show somewhat less bright polar regions as compared to the ultraviolet. Digital filtering or removal of scattering geometry effect (normalization) using a scattering law brings out subtle contrasts on very small scales (~ 10–20 km) at 0.9 and 2.02 µm.

Given the prevalence of submicron (Kawabata et al. 1980; Luginin et al. 2016) haze above the clouds in the 70–90 km region and ~ 1.1 µm effective radius of the cloud particles, the lack of detail in the images is consistent with the large degree of homogeneity of these particles on the dayside. However, there are known to be some larger particles in the deeper layers, so any features that can be seen may be indicative of the presence of larger particles at higher altitudes. The brightness normalized versions do show some contrast features whose appearance is quite different from that seen at 283 and 365 nm. The two processed versions (b and c) of image a show patterns that are not readily identifiable in the calibrated version—an elongated “S” shape in the top center of the image. Toward the bright limb on the right, a curved bright feature is seen in the filtered version, but its shape is somewhat different in the normalized version. An almost straight, dark and narrow line is seen in the lower right quadrant of the image, inclined at almost 45**°** with the equator. In versions e and f of the calibrated image shown in d, the appearance of the features is markedly different. A long, linear feature is seen just north of the equator at a small angle, while a bow-like feature is seen in mid-northern latitudes whose orientation is almost orthogonal to the bow-shaped features seen in the ultraviolet images. Patterns such as these are not generally visible in the ultraviolet images. The normalized version of image g shows a large dark patch north of the equator which is trailed by curved features in the northern hemisphere (bottom half of the image), while a linear feature is seen in the south. The patch is located between about 10°N and about 43° latitude (lower right portion of the image) with a trailing narrow dark band toward the bright limb (left) beginning at the eastern edge of the patch at about 25°N latitude. The appearance is somewhat like a contrast reversed version of the Y, but is quite different from the near simultaneous 365-nm image (see Fig. 11). Quantitative comparison of the 09d image intensities with other wavelengths is pending as the calibration of the data is improved.

#### Images at 2.02 µm

**Fig. 8:**
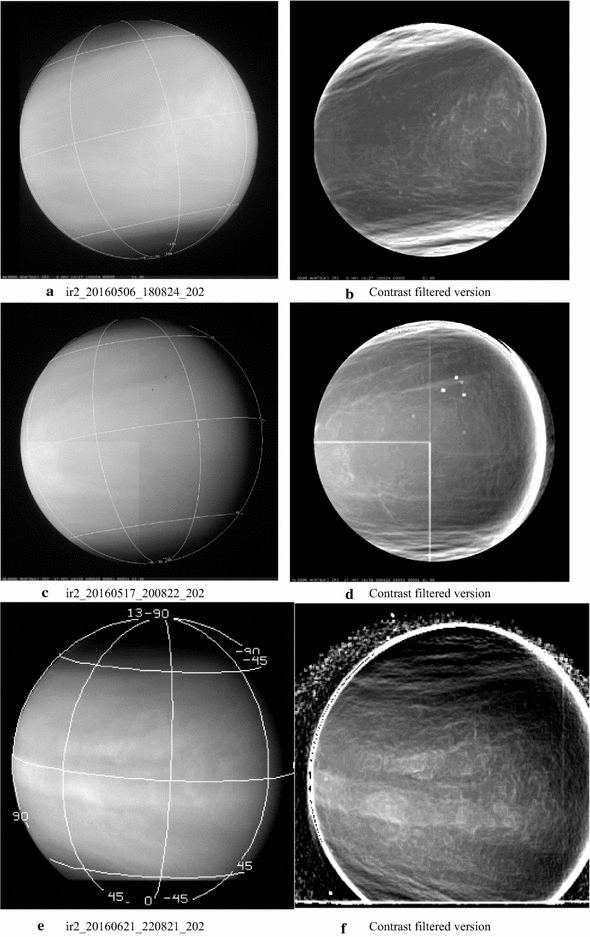
A sample of full-disk dayside 2.02-µm images from the IR2 camera. The calibrated versions are shown in the left column and contrast filtered versions are shown in the right column to bring out the very low-contrast details present in the data. At this wavelength, the absorption due to CO_2_ is noticeable at polar latitudes where the cloud tops are lower, resulting in lower brightness values. Although the observed cloud features are believed to be at the same altitude range as those seen at 283, 365 and 900 nm, different morphologies are seen. For example, the image shown in **c** shows a bright band, which is more noticeable in the filtered version (**d**), much like the “belt” seen on Jupiter, but is not visible at the shorter wavelengths. The contrast filtered versions and normalized version of the images also show features not seen in the shorter wavelengths. The cause of these differences has not yet been explored

Full-disk dayside 2.02-µm images from the IR2 camera are shown in Fig. 8. Again, both the calibrated (a, c and e, left column) and filtered versions (b, d and f, right column) are shown, but the filter is different from that used for the IR1 images shown in Fig. 1. Here, a custom “contrast” filter is applied which uses a scaled ratio of the standard deviation to the average brightness of all of the pixels in a moving box (11 × 11 pixels) and is centered at each pixel within the region of the image away from the outer boundaries by half the dimension of the box. For cosmetic reasons, a fraction of the original image is also added back. The polar regions (> 60**°** latitude) appear noticeably darker at this wavelength close to the equatorial boundary of the “cold collar” seen in the thermal data (images a, c and e). At this wavelength, some of the radiation reflected from the clouds is absorbed by CO_2_, the dominant constituent of the Venus atmosphere. The darker regions thus suggest lower cloud tops, which would be consistent with the lower cloud tops inferred from VIRTIS data at 1.6 µm using the same approach by Ignatiev et al. (2009). The equatorial and midlatitude regions show long, relatively thin string-like features, somewhat similar to those seen at the ultraviolet wavelengths, suggesting some altitude relief. These features are more visible in the contrast filtered versions (b, d and f). The classic Y-shaped feature has often been discerned at 2.02 µm in the global views, suggesting some correspondence between the mechanisms or processes creating or contributing to the contrasts seen at the ultraviolet and at 2.02 µm (image b). There also appears to be some phase angle dependence in the contrasts as very few details are visible at phase angles > 120**°**. Image d reveals a “belt”-like bright band about the equator, which is unusual and is better seen in the contrast filtered version. Smaller-scale curved features suggestive of local circulation are seen in image d in the bright region toward the left of center of the frame, which have not been discerned at the shorter wavelengths on the dayside. Since the contrast filter adds back a fraction of the original image, the increased brightness of this portion is due to the scattering geometry.

### Morphology at smaller scales (~ 2 to ~ 5 km/pixel)

**Fig. 9:**
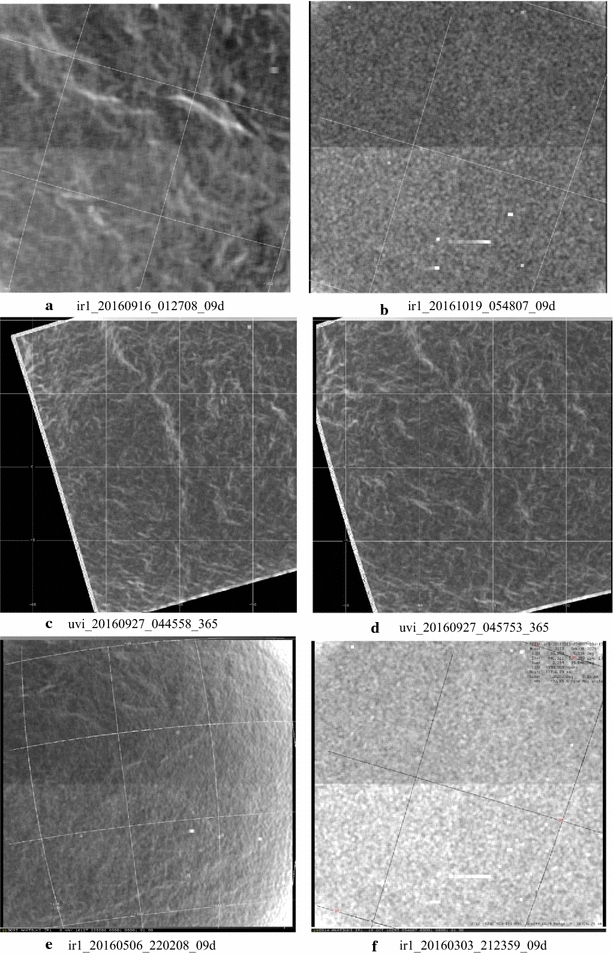
High-resolution images from the IR1 camera (top and bottom rows) in the 09d filter and two UVI images taken through the 365-nm filter (**c** and **d**, middle row). Only contrast filtered images are shown to emphasize the details. The UVI images are taken about 12 min apart and are shown as rectilinear maps to show the rapid changes that can take place in the appearance of the wavy features. In the 0.9-µm images **a**, **e** wavy and curved patterns are also seen; however, the slightly higher-resolution images **b**, **f** do not show such details. It is not known whether this is due to the transient nature of the patterns or due to spatial-scale dependence of contrasts

The small-scale contrast features have a different morphology from the large-scale features seen in global images. VMC images taken at all four wavelengths (365, 550, 950 and 1050 nm) showed fine-scale (1–5 km spacing) gravity waves of four distinct morphologies (long, medium, short and irregular) in the high northern latitudes (Piccialli et al. 2014) which were not previously detected and have not yet been seen in Akatsuki images at any wavelength. Images at ~ 2 to ~ 5 km/pixel scale are generally acquired by the Akatsuki cameras only at low latitudes, and thus, the high-latitude fine-scale gravity waves cannot be seen; however, the images do show some surprising structures. Figure 9 shows a selection of views from the UVI and IR1 cameras. Only contrast filtered versions are shown as the calibrated images show barely discernable details. Images c and d are mapped at 0.025° per pixel in latitude and longitude to present the same scale since the two images were taken only about 12 min apart and thus show that the features change rapidly at the small scales over a few minutes. Both the images shown in c and d show the equatorial region and span an area approximately 400 × 400 km. On this small scale, the orientation of the bright streaks at 365 nm is almost orthogonal to the latitude circles, unlike the general pattern of streaks (a fixed source region, e.g., the linear features seen in d) observed over larger scales which are inclined at a smaller angle, giving the impression that they are streak lines (Smith and Gierasch 1996; Suomi 1975). Whether these streak-like features seen in c and d are streak lines is not yet known.

**Fig. 10:**
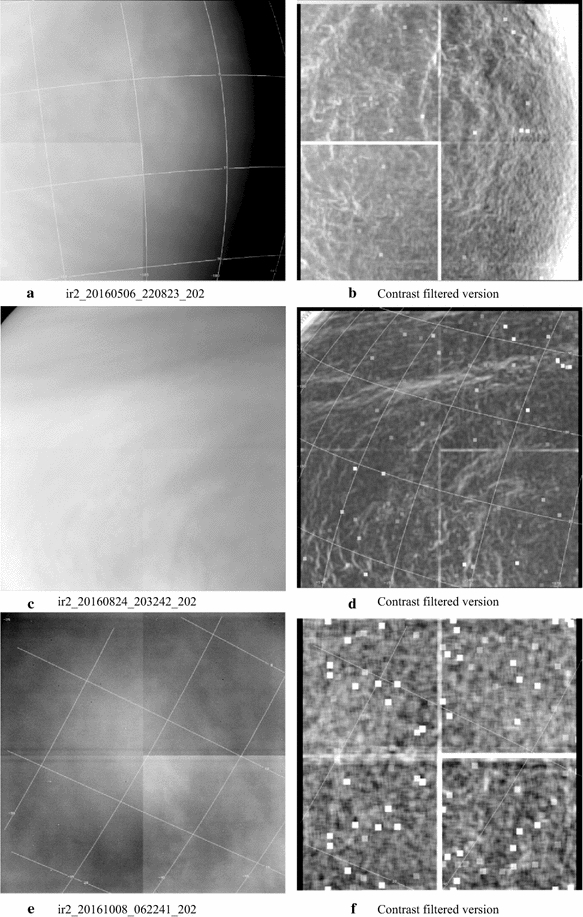
A sample of high spatial resolution dayside 2.02-µm images from the IR2 camera, each at a pixel scale of approximately 5 km in the calibrated (left column, **a**, **c**, **e**) and contrast filtered versions (right column, **b**, **d**, **f**). The quadrant boundaries of the CCD can be faintly seen in the calibrated version due to slightly different gains of the readout electronics. Very bright pixels in the contrast filtered versions represent noise pixels. Very subtle sinuous or string-like structures are seen with widths of about 20–40 km and with variable lengths and inclinations to latitude circles as seen in **a** and **b**. Image **b** shows bow-like waves also seen at ultraviolet wavelengths. Image **e** is devoid of such patterns, but instead shows a bright area surrounded by a poorly defined dark ring, which results from low-frequency electronic noise due to the cross talk between the readout electronics of the four quadrants of the image. This pattern disappears in the contrast filtered version (**f**). It is interesting that the thin wavy streaks seen in **b** and **d** are absent in **f** which has almost twice the spatial resolution, similar to the difference at the shorter wavelengths (images **b** and **f** in Fig. 9)

Figure 10 shows higher-resolution 2.02-µm images in both calibrated and contrast filtered versions. In general, the contrasts are even lower on such small spatial scales and are brought out only after some filtering. Images a and b show thin, string-like features of variable lengths, curvature and inclinations to latitude circles. Image e (and a filtered version in f), however, is surprisingly devoid of such features but shows a bright region core surrounded by a dark ring, like the classic signature of the glory feature which has been seen in the Venus Monitoring Camera at 365, 550, 950 and 1050 nm (Markiewicz et al. 2014). However, the geometry of this image is not consistent with the backscatter angle and the feature is believed to be due to low-frequency noise and cross talk between the four quadrants of the IR2 readout electronics (Satoh et al. 2017). For lower-resolution images which contain a lot more detail, this low-frequency noise is not a significant hindrance in the interpretation of the images, but its removal certainly improves the image appearance somewhat as shown by Satoh et al. and is necessary for quantitative comparative studies.

### Contemporaneous views at 283, 365 nm, 1.02 and 2.02 µm: spatial variations in cloud properties

**Fig. 11:**
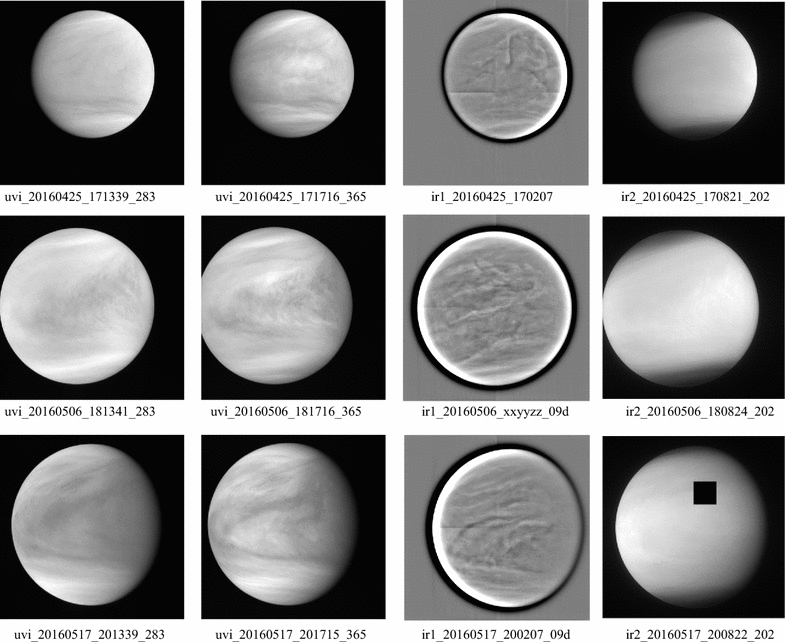
Concurrent views of the dayside hemisphere of Venus at 283, 365, 900 and 2020 nm. The 900-nm images are shown after applying a high-pass filter to bring out subtle detail. The high-latitude regions appear darker at 2020 nm due to the increased absorption by CO_2_, indicating somewhat lower cloud tops. The black square in the May 17 IR2 image is due to data dropouts during telemetry

Near simultaneous global images of Venus at 283, 365 nm as well as 0. 9 and 2.02 µm on three separate days (almost one orbit apart) are shown in Fig. 11. The 0.9-µm images are shown in filtered versions to bring out the contrasts. The transition between lower and higher brightness matches well at 283 and 2.02 µm, while the transition occurs at a slightly lower latitude at 365 nm. A bright streak or band usually marks this transition in the 283-nm images. Poleward of this, quasi-linear streaks can be seen at all four wavelengths which are equivalent to the spiral arms of the vortex over the pole which can be better seen in polar projections presented later. The two longer wavelengths should probe the deeper cloud levels as compared to the ultraviolet wavelengths as they are less affected by scattering due to the smaller particles, but at 2.02 µm, CO_2_ absorption also contributes to the depths probed. Thus, when similar features or structures are seen at all four wavelengths, the features extend from near the top to at least a scale height deeper and should indicate the presence of larger particles. When the features are not similar, the cloud cover may be due to vertical variation of the cloud particle properties in the upper cloud.

**Fig. 12:**
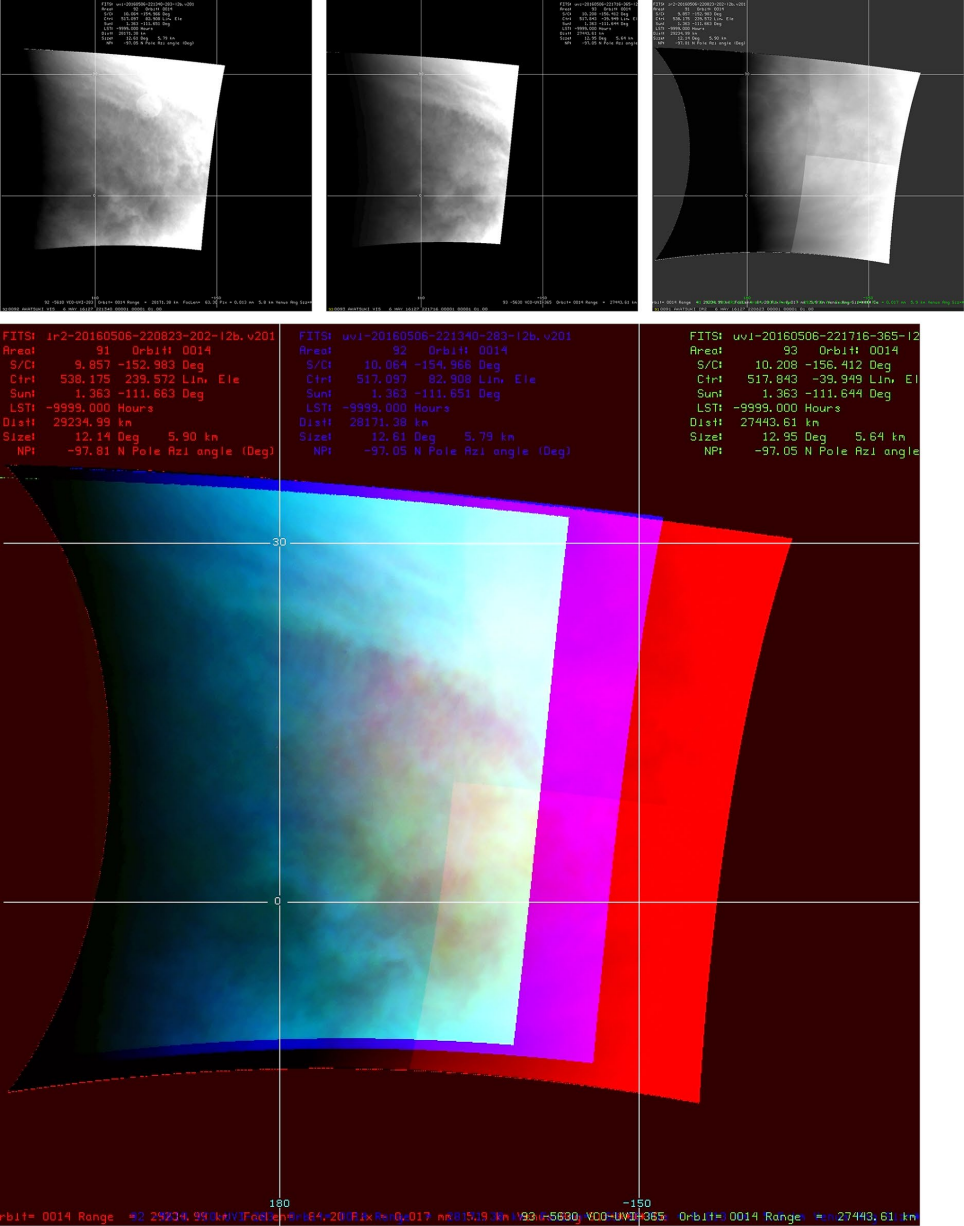
High-resolution views of Venus at 283, 365 and 2.02 µm (~ 6 km per pixel) from the Akatsuki UVI and IR2 cameras on May 6, 2016, are shown in the upper row. A color composite view of these images is shown in the lower row and is created from 2.02-µm (red), 365-nm (green) and 283-nm (blue) filter images. The oval-shaped bright spot seen in the 283-nm image is an artifact of the 283-nm filter. The quadrant boundaries of the CCD detector of the 2.02-µm camera can be seen in the image shown (top row, right column) due to unmatched gains of the readout amplifiers. Contrasts are seen on scales of about 100 km at 283 and 365 nm, and perhaps on a slightly larger scale at 2.02 µm; however, this may be due to the lower level of contrasts and the dynamic range of the data

Figure 12 shows images obtained close together in time and at a spatial scale of about 6 km per pixel at 283, 365 nm and 2.02 µm in the top row mapped in latitude and longitude. The lower column shows a color composite with 283 nm shown in blue, 2.02 µm in red and the 365-nm image in green. Subtle color variations over the overlapping area from yellow to purple or brown are suggestive of differences in the distribution of submicron haze (~ 0.2 µm radius), the cloud particles (~ 1.2 µm radius), larger particles and possible differential CO_2_ amounts over the “cloud tops.” More bluish and/or greenish portions indicate smaller abundances of absorbers, while reddish shades indicate a higher cloud-top level over other regions due to less CO_2_ absorption above the cloud top in the CO_2_ band (2.02 um). Figure 12 shows yellowish areas in the 10°S–10°N latitude, implying the area has less unknown absorber(s) and the cloud-top level is higher than the surrounding region. This may be related to local circulations caused by differential heating from variations in the abundances of the absorbers on the dayside.

## Nightside morphology

On the nightside, the IR2 and LIR cameras of Akatsuki probe different depths of the Venus cloud layer as compared to the dayside images. The LIR camera (8–12 µm) probes the 65 km level and provides cloud-top temperature variations over the planet on day and night hemispheres.

At 1.74, 2.26 and 2.32 µm wavelengths, the features seen in the images can be understood as being back-lit silhouettes illuminated by the radiation emitted from the increasingly warmer atmosphere below the clouds. The dayside features are due to differences in either the amount of absorbers and/or cloud altitudes and inform us about the cloud-top region, while on the nightside, the features are due to the spatial variations in the emitted radiation from the atmosphere below the clouds as it gets attenuated by the cloud opacity in the atmospheric windows at 1.74, 2.26 and 2.32 µm (Wilson et al. 2009). Thus, the features do not necessarily reflect a fixed level in the cloud layer. This is analogous to the appearances of Jupiter (Orton et al. 1991) and Saturn at 5 µm (Orton and Yanamandra-Fisher 2005). It can be expected that the morphologies seen in the IR2 and LIR images will be somewhat different. We describe the nightside morphology from IR2 images first and then the LIR images next.

### 1.74-, 2.26- and 2.32-µm images from IR2 camera

**Fig. 13:**
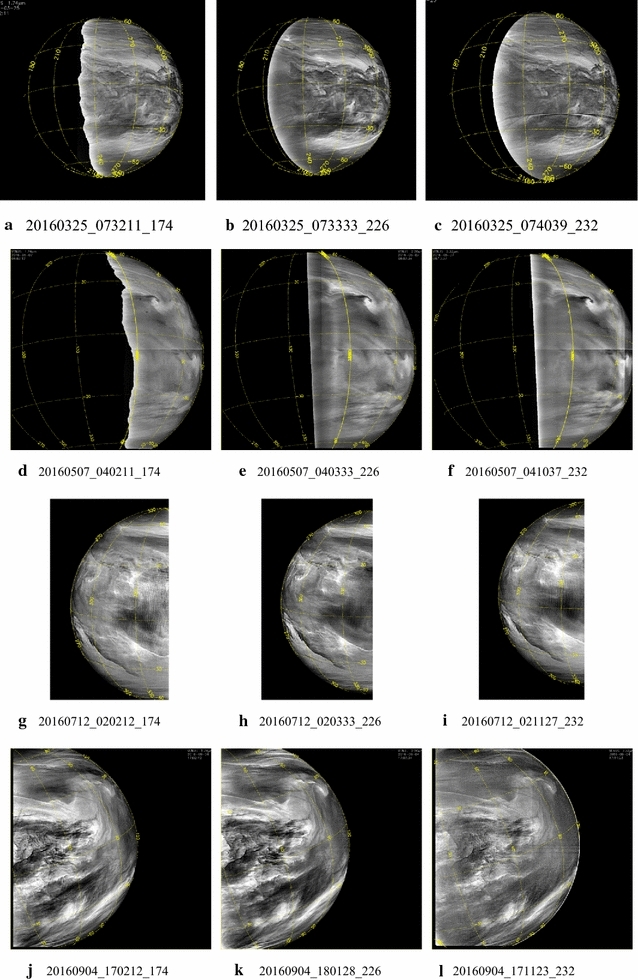
Selected images at 1.74, 2.26 and 2.32 µm taken from the IR2 camera on the nightside of Venus. To bring out the intricate details, the images have been filtered using a combination of unsharp mask and adaptive histogram equalization

**Fig. 14:**
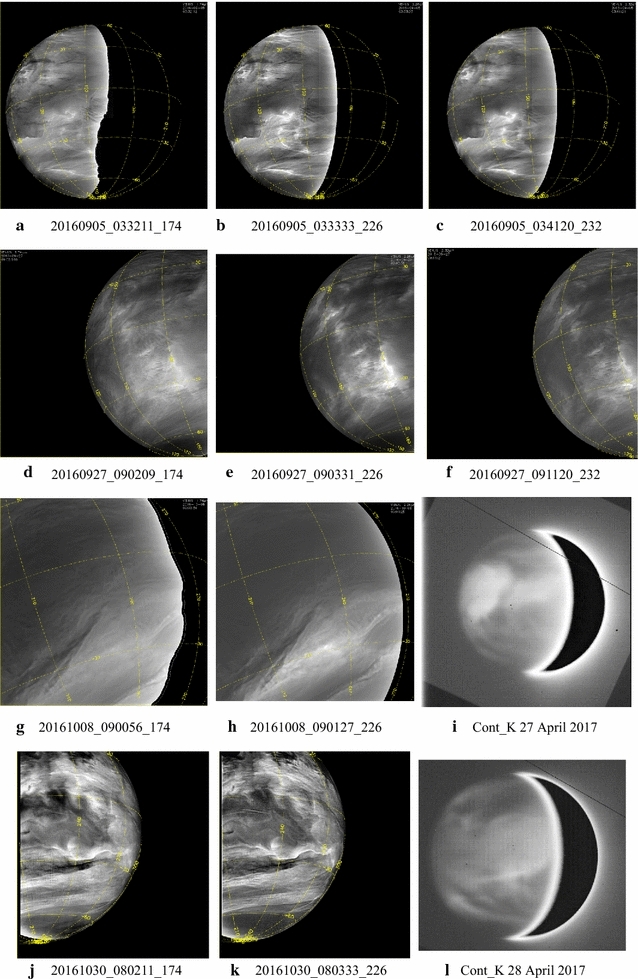
Selected images at 1.74, 2.26 and 2.32 µm taken from the IR2 camera on the nightside of Venus (except **i** and **l**). To bring out the intricate details, the images have been filtered using a combination of unsharp mask and adaptive histogram equalization. Images **i** and **l** were taken from the NASA Infrared Telescope Facility (IRTF) on Mauna Kea, Hawaii, as part of the ground-based imaging campaign in support of the Akatsuki mission. Compared to the dayside, features seen in these nightside near-infrared images are very different and show a large variation in patterns

Figures 13, 14 show a selection of nighttime images taken from the IR2 camera at 1.74, 2.26 and 2.32 µm which reveal a very different morphology than that seen on the dayside. All IR2 nightside images have been processed with a combination of unsharp mask and adaptive histogram equalization in order to emphasize the local details of the cloud opacity structure. Also included are two sample images (14i, l) from the NASA Infrared Telescope Facility (IRTF) located at Mauna Kea, Hawaii, through the 2.26-µm filter (K-continuum). These two images are part of a ground-based campaign to collect supplemental images in support of the Akatsuki mission. Although the NIMS (Galileo) and VIRTIS (Venus Express) instruments also provided images at these wavelengths (Carlson et al. 1991), the coverage was limited due to the flyby nature of Galileo observations for studying global morphology. VIRTIS obtained only limited coverage of the low latitudes and could not provide global views of the planet from an equatorial perspective due to the long integration time required for the spectral cubes and the elongated, polar orbit of Venus Express.

The features seen in the IR2 images suggest that dynamics plays a significant role in the nightside morphology. Some latitudinal differences are apparent. At higher latitudes, the features are more subdued, while the low latitudes are full of small- and large-scale features. Images 13a, b, c show a myriad of wavy, linear, patchy and chevron-shaped features. Images 13d, e, f show mushroom-shaped mesoscale vortices and few features in low latitudes. In contrast, 13g, h, i, j, k, l show an abundance of detailed structure on difference scales and in formation. For example, the lower left portion of the images g, h and i of Fig. 13 shows a long linear dark streak (low intensity) at some angle to the latitude circles as well as a very rare bright small region at 1.74, 2.26 and 2.32 µm (high intensity) near the center of the images. An almost parallel bright streak is seen to the upper right of the dark streak in all three filters in images g, h and i. Some of the parallel, evenly spaced circular arcs seen in many of the images shown in Figs. 13 and 14 are likely artifacts of digital filter processing. The differences in morphology seen in images 13j, k, l, and 14a, b, c are striking as they depict the evening and morning portions of the nightside hemisphere as the spacecraft moved past periapsis. Whether this is a purely local time effect or a transient phase is not yet known for lack of sufficient coverage of the morning and evening regions over a short period which occurs only when periapsis passage takes place on the nightside of Venus. Image triplets 14a, b, c, d, e, f taken about 3 weeks apart show features, which have similar appearance. Eleven days after the d, e and f triplet, the appearance of Venus is markedly different (images 14g, h). The two IRTF images have lower spatial resolution but show features which differ from those in some IR2 images, such as a large feature oriented northwest–southeast in 13i and 14l. Image 14i resembles the 14a, b, c triplet with features showing sharp angles and intensity boundaries, while features in image 14l resemble those in the 13g, h pair.

At high spatial resolution all three wavelengths show bright, thin, sinuous wispy streak-like features against a darker (lower-intensity) background (images 14d, e f). Such features have not been detected on the dayside at any wavelength in the images obtained thus far. Often these features are seen to crisscross, perhaps suggesting some altitude difference analogous to terrestrial cirrus, but we have little additional data or information at this time. Regardless, their presence is probably due to some cloud particle formation process in the lower or mid-cloud layer which is affected by trace species, condensation nuclei and temperature. Volcanic or intense surface outgassing plumes can provide cloud condensation nuclei and trace species that lead to cloud formation as well.

Different processes may be producing a very high linear opacity feature in the case of the dark streak and a much more localized “hole” in the overlying cloud to let the near-infrared radiation leak through from the lower atmosphere to space. The localized increase in radiance can also be due to somewhat elevated temperatures in the lower atmosphere, but a better understanding can come only from modeling the cloud structure.

### Sharp boundaries: Different air masses?

Some nightside IR2 images show a sharp linear boundary running roughly north–south across which the intensity varies noticeably (e.g., images 13j, k, l as well as 14a, b, c). What is remarkable is the sharpness of the boundary with changing orientation. Origins of such sharp opacity regions are not easy to explain dynamically without invoking “different air masses” as was suggested to explain the VeGa balloon dynamical results (Blamont et al. 1986), but then the challenge is explaining the origins of different air masses.

**Fig. 15:**
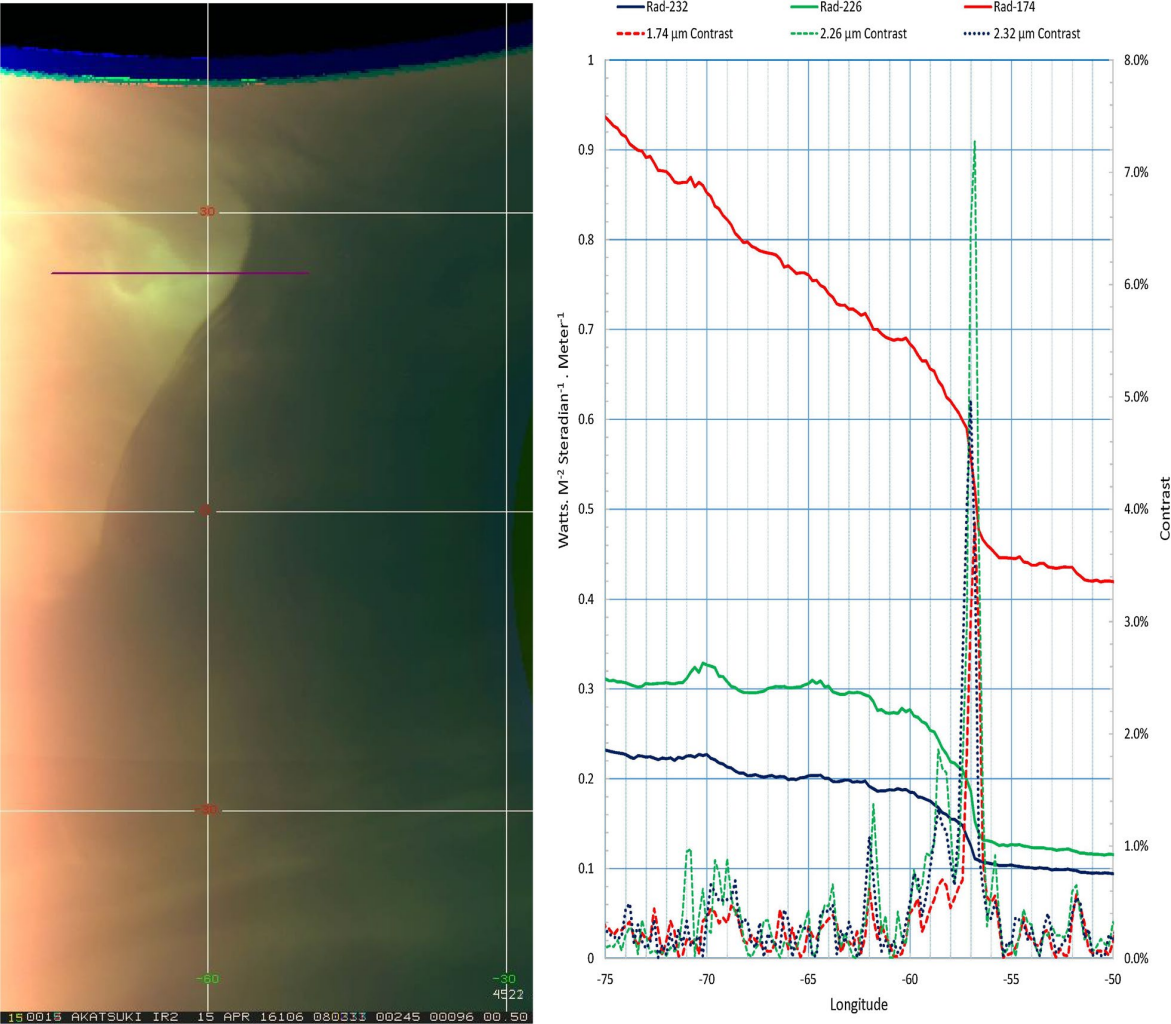
A latitude–longitude color composite map of 1.74-, 2.26- and 2.32-µm nightside images obtained from the IR2 camera on April 25 showing an unusual sharp boundary in the intensity (left). A latitude–longitude grid (30° spacing) is overlaid with the equator along the middle of the image. On the right are plots of radiance and the local contrast at three wavelengths along 24°N latitude, from − 75° to − 50° longitude (marked on the color composite) that cuts across the “mushroom” feature and the sharp boundary that occurs at about − 56° longitude. The contrast is defined here as the difference in radiance at the two neighboring pixels of a given pixel divided by the average of their two pixels. Such sharp boundaries are seen on Earth only in water vapor channels, thereby showing the dry and moist air masses (Fig. 16)

**Fig. 16:**
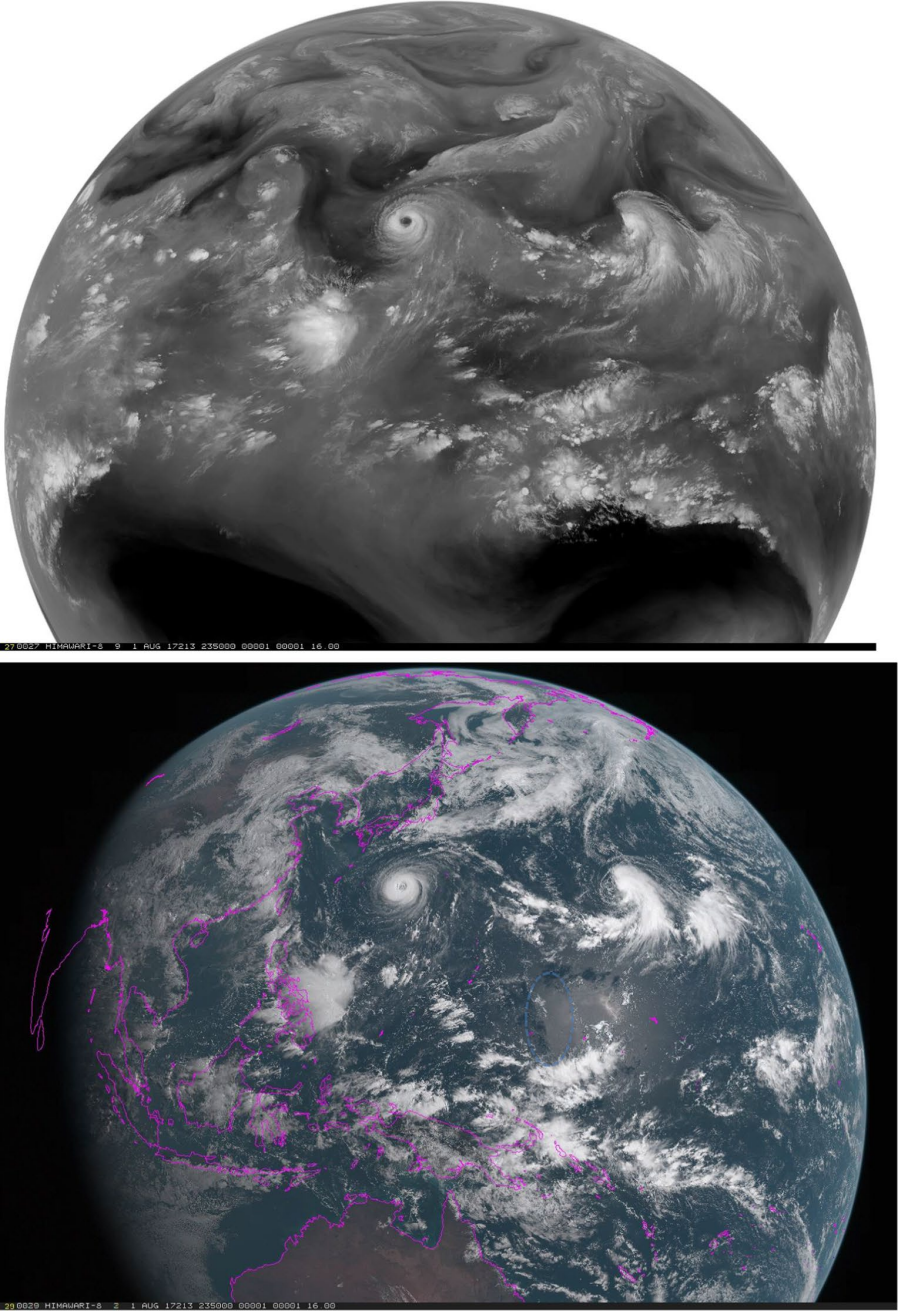
Sharp boundaries are also seen in images of Earth taken from weather satellites in the water vapor channel (6.9 µm channel top) and a color composite of three visible channels from Himawari on August 1, 2017, at 23:50 UT. Several sharp boundaries in the water vapor channel can be seen in both hemispheres in the vicinity of Typhoon Noru and the cyclonic circulation further east, and in the southern hemisphere due to different air masses with different lower tropospheric water vapor amounts. The bottom image is a red, green and blue composite view of three images taken simultaneously through different visible filters where the boundaries between the dry and moist air masses are not so easily discerned but can be inferred from the destruction of the clouds due to entrainment of the dry air in the western half of the cyclonic region (central part of the color image). Note also a boundary just west of the brighter Sun glint in the color composite to the right of the frame center which is not seen in the water vapor channel due to variable sea roughness across the boundary. By analogy, the sharp boundaries seen on Venus in the IR2 nighttime images may suggest different “air masses” due to differing trace gas abundance(s) within the Venus cloud layers

Figure 15 shows an example with a very striking intensity boundary which is seen at 1.74, 2.26 and 2.32 µm, at the same location and cutting across latitude circles from north to south around − 60° longitude. The right panel shows the radiances at 1.74 (red), 2.26 (blue) and 2.32 (green) µm across this boundary. The change in the radiances across the boundary is also shown as a percent contrast (difference of neighboring pixels divided by the average brightness of the neighbors at each pixel along the scan) and is a few percent across the boundary at all three wavelengths. The boundary appears analogous to a frontal zone separating a moist air mass from a dry air mass on Earth and also shows some fine structure presented by Satoh et al. (2017). This boundary remains more or less intact for some time—between 2 and 10 days before dissipating, much as the expanding plume from a volcanic eruption or a dust storm front evolves on Earth. The duration of the sharp boundary seen in the IR2 images is uncertain due to lack of images as the sharp boundary region rotates out of the field of view in the intervening days. On Earth, sharp boundaries are also seen in water vapor channel images (Fig. 16), revealing different mid-low tropospheric water vapor abundances. The right angle between dry (dark) and moist (gray) shown in Fig. 16 southeast of Japan is similar to the feature in images a, b and c in Fig. 14. Although trace species such as water vapor, HF and CO are optically active in the 2.3 µm region and can be responsible for such discontinuities, the causes for such horizontal demarkation in the species abundances are unclear. Since such boundaries are not apparent in the dayside, the differences in the abundances may be restricted or relevant in the middle and lower part of the cloud, perhaps due to the increasing stability of the atmosphere with altitude. Magurno et al. (2017) have examined VIRTIS data between 25° and 55° latitude to explore cloud particle properties and trace species and find that sulfuric acid concentration making up the cloud particles may be varying vertically and that near 2.3 µm, water vapor, CO and COS affect the observed intensities (CS_2_ was not included). Regional dynamics must therefore be responsible to create the observed sharp boundaries. If water vapor is responsible for the sharp boundary, then the question is how different air masses come about on Venus, which is not a problem on Earth. Volcanic eruptions can be speculated as a cause, but as yet we have no corroborating evidence.

Images g and h in Fig. 14 also show a similarly sharp boundary, but in this case, the low-intensity region is shaped like a dark streak. Ground-based IRTF images (i and l) also show large contrasts in the intensities in the K-continuum filter.

Interestingly, the normalized and high-pass filtered versions of one image taken on the dayside (Fig. 7, image a) also show a boundary. No such boundaries have yet been detected at the ultraviolet wavelengths. We suspect that the local dynamics must play a role in affecting the distribution of aerosols at the levels on both day- and nightsides involved for lack of any other evidence. These features serve as a caution against basing interpretations of the cloud patterns using the VIRA model (Kliore et al. 1985) since local deviations from local circulations are clearly significant. At this point, it is premature to assess the impact of these local opacity variations on the heat deposition in the atmosphere which is believed to be driving the superrotation of the deep atmosphere of Venus.

### Mesoscale vortices

Mesoscale, local circulations including vortices with spatial extent of ~ 1000 km are also seen in a few of the images for the first time on Venus (d, e, f in Fig. 13) which resemble “mushroom”-like features seen in water vapor images of Earth from synchronous weather satellites (Houghton and Suomi 1978). These features are further discussed by Limaye et al. (2017). The origin of such smaller-than-planetary-scale circulations was not expected due to the low rotation rate which places Venus in a “symmetric” circulation regime rather than a planetary wave regime as was discovered by Hide (1953) and Fultz from dish pan or rotating annulus experiments to simulate atmospheric circulations many decades ago, and analyzed extensively since (Read 2011; Sugata and Yoden 1993). Their appearance suggests a vorticity generation process perhaps due to the vertical shear of the zonal winds and vertical motion. Their lifetimes are uncertain, but could be as long as a month based on two sightings and without the confidence that it is the same feature. More observations and detection of such vortices would be very useful, but orographic origins are suggested. A search in the VIRTIS data shows some similar features.

### Ribbons or narrow width waves

**Fig. 17:**
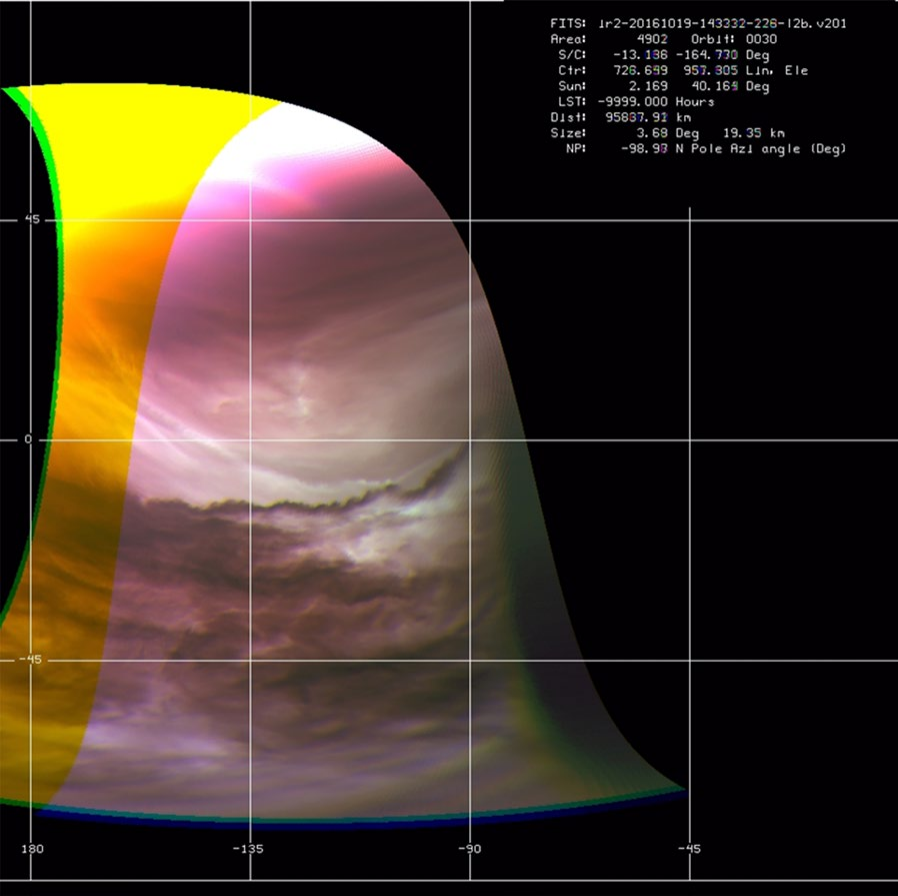
Images taken on 19 October from the IR2 camera show a dark wavy feature at 1.74, 2.26 and 2.32 µm and mark a boundary between cloud masses to the north and south at some unknown altitude. A color composite of these images in rectilinear projection is shown here. The three filters do not completely overlap due to small differences in the times when they were acquired and the orbital motion of Akatsuki

A sinuous dark ribbon has been seen along a latitude circle with a width of ~ 500 km and with wavelength varying from ~ 800 to 1200 km in one 2.32-µm image (Fig. 17) that separates a darker region toward the equator and brighter region south of it toward the pole. Such a sharp transition is generally seen at the short wavelengths on the dayside but between 45° and 50° latitude in both hemispheres, and generally without a sinuous nature (Rossow et al. 1980; Suomi and Limaye 1978; Titov et al. 2012). Figure 12 shows a color composite of a 1.74-, 2.26- and 2.32-µm image triplet in a rectilinear format (to enable registration of the three frames) from the IR2 camera (left) and in a latitude–longitude view (right). The wavelike boundary is at about − 17.3° latitude. The larger intensities at higher latitudes suggest thinner (lower opacity) overlying clouds. Less prominent waves have also been noticed at other latitudes but without opacity transitions, both with IR2 and Venus Express/VIRTIS (Peralta et al. 2008). It is not yet known what the different patterns imply regarding the dynamical processes and opacity differences.

### High-resolution detail on the nightside

**Fig. 18:**
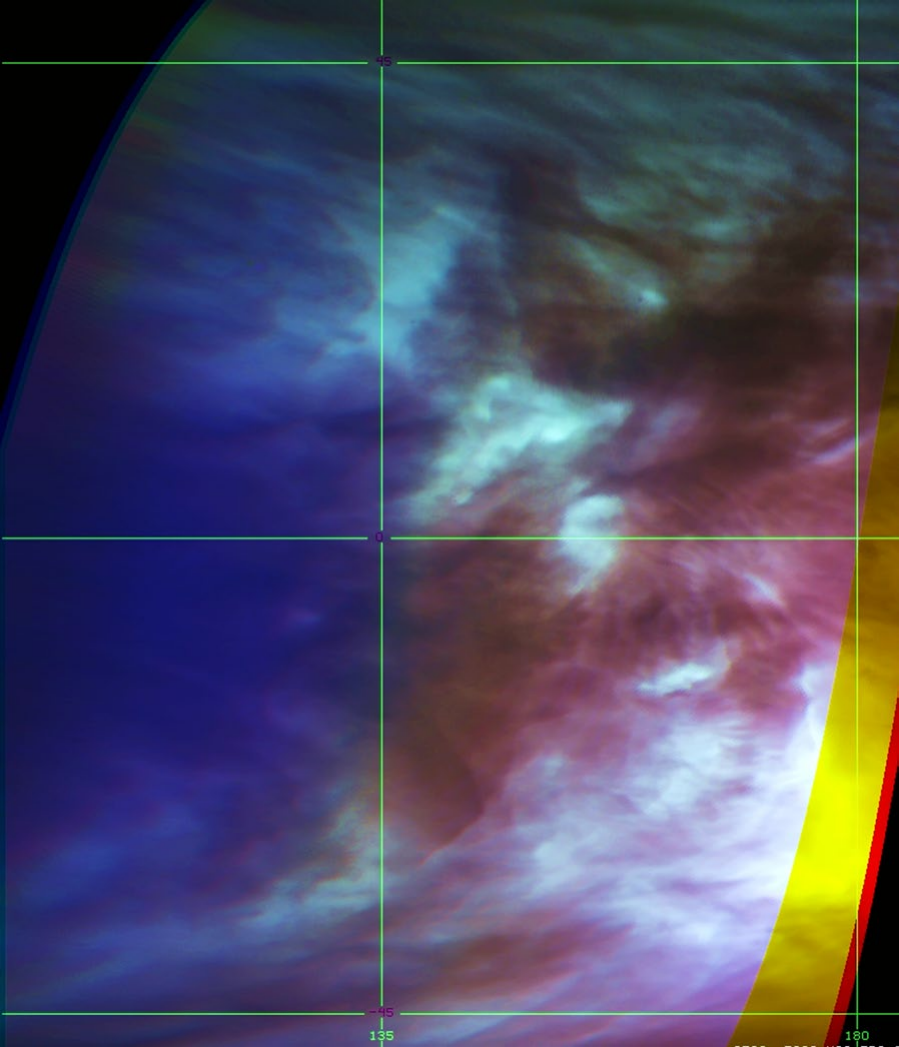
Subtle variations in the opacities are revealed in this color composite generated from mapped 1.74-µm (red), 2.26-µm (green) and 2.32-µm (blue) images taken on September 27, 2016, at 11:02:09, 11:03:31 and 11:11:19 UT, respectively. The image scale is 0.1°/pixel in latitude and longitude. The images have not been corrected for any limb darkening but are calibrated. The color variations illustrate the variation of opacities at the wavelengths arising from trace species and aerosol abundances

Figure 18 shows a color composite of colocated 1.74-µm (red), 2.26-µm (green) and 2.32-µm (blue) images obtained from the IR2 camera at a spatial scale of ~ 11 km/pixel. All three images are shown as latitude–longitude maps with a 0.1°/pixel scale. Color differences reveal subtle differences in opacity of the Venus clouds at the three wavelengths in this color composite. Thus, white features having similar opacity values and different colors indicate higher or lower values of emitted radiance and are attenuated by overlying atmospheric and cloud opacity. Assuming no emission differences, areas that appear yellowish in the overlap region should indicate somewhat higher opacity at 2.32 µm as compared to the other two wavelengths, and the reverse in areas where the features appear somewhat bluish. These differences appear consistent with influences of CO contribution at 2.32 µm (Tsang et al. 2009), H_2_SO_4_ absorption (1.74 vs. 2.2 µm (Barstow et al. 2012) and cloud particle size (1.74 vs. 2.3 µm, Wilson et al. (2008)) and have been identified from Venus Express data. It seems that the compositional differences do not appear to be due to local time differences.

### Thermal infrared: cloud-top brightness temperature morphology from LIR

The Pioneer Venus OIR experiment provided the first spacecraft observations of Venus at thermal wavelengths where the emission from its cloud tops can be sensed (Taylor et al. 1980); however, these were limited to northern polar regions. The Akatsuki LIR camera provides the first continuous global views of the brightness temperature distribution over the cloud cover. The camera samples radiation between 8–12 µm (Taguchi et al. 2007) and the weighting function peaks at 65 km (Taguchi et al. 2007) with a half width of ~ 10 km for the nominal Venus cloud structure (Knollenberg and Hunten 1979). The LIR band pass includes the SO_2_ absorption band at 8–9 µm and also the CO_2_ absorption band between 9.5 and 10.4 µm. Thus, it may be important to account for the potential contributions by these two gases due to the abundance variation of SO_2_ or cloud-top altitude variations, which can impact the CO_2_ contribution to the brightness temperature sensed by LIR.

### North–south stationary wave

**Fig. 19:**
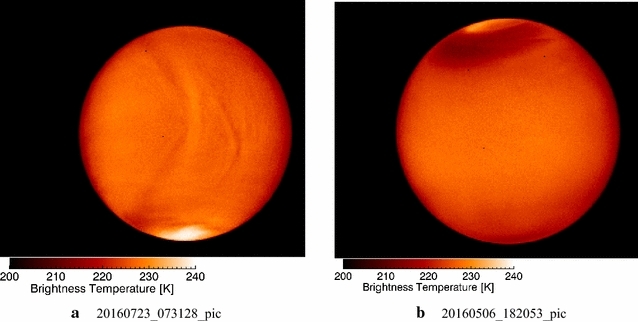
Representative images of Venus taken by the LIR camera at 8–12 µm. **a** Standing gravity waves discovered by Akatsuki from such observations (Fukuhara et al. 2017a, b), **b** Venus when the standing wave is not present. Generally, the equatorward edge of the vortex over the poles is seen in all of the LIR images

Figure 19 shows two sample images taken from the LIR camera. One of the first major discoveries from Akatsuki from the very first image obtained by LIR was a bright band (image a in Fig. 19) aligned almost north–south with a slight curvature which has been interpreted as a standing gravity wave triggered by surface topography (Fukuhara et al. 2017a). Such waves have often been seen in Akatsuki LIR data and it has been observed that the north–south linear or bow features are seen as stationary at certain local times (3:00–7:00 PM) in the LIR data (Fukuhara et al. 2017a). Further analysis of LIR data shows that these waves last as long as 2–3 weeks. These waves have a brightness temperature contrast of about 5–6 K with the bright and dark portions corresponding to about 230 and 225 K, respectively. Surprisingly, the signature of these standing waves can also be detected at 283 nm (Fukuhara et al. 2017a) and at 365 nm (less frequently compared to 283 nm) and in 2.02-µm IR2 dayside images, also after filtering (Satoh et al. 2017). The detection at 2.02 µm suggests that cloud-top variations likely occur due to the wave. Surprisingly, no stationary waves have yet been detected in 0.9-µm daytime images from the IR1 camera and they also have not been detected in MESSENGER MDIS images or VIRTIS data at this wavelength. A north–south pattern resembling the stationary wave (a bow shape with little curvature) was also seen in the 986-nm images of Venus from the Galileo SSI (120°–180°E), but was not identified as caused by topography and was described only as a north–south brightness discontinuity moving with the ambient wind by Belton et al. (1991).

Except for the presence of such standing waves, the cloud cover shows very small brightness temperatures over most of the planet (image b in Fig. 19) of less than 2 K. Only at polar latitudes are larger brightness temperature variations seen. The “cold collar” often can be identified as a darker ring surrounding the warm core region of the vortex over the pole, which appears bright due to emissions from the deeper atmosphere.

### Global organization of the cloud cover: vortex situated over the poles

**Fig. 20:**
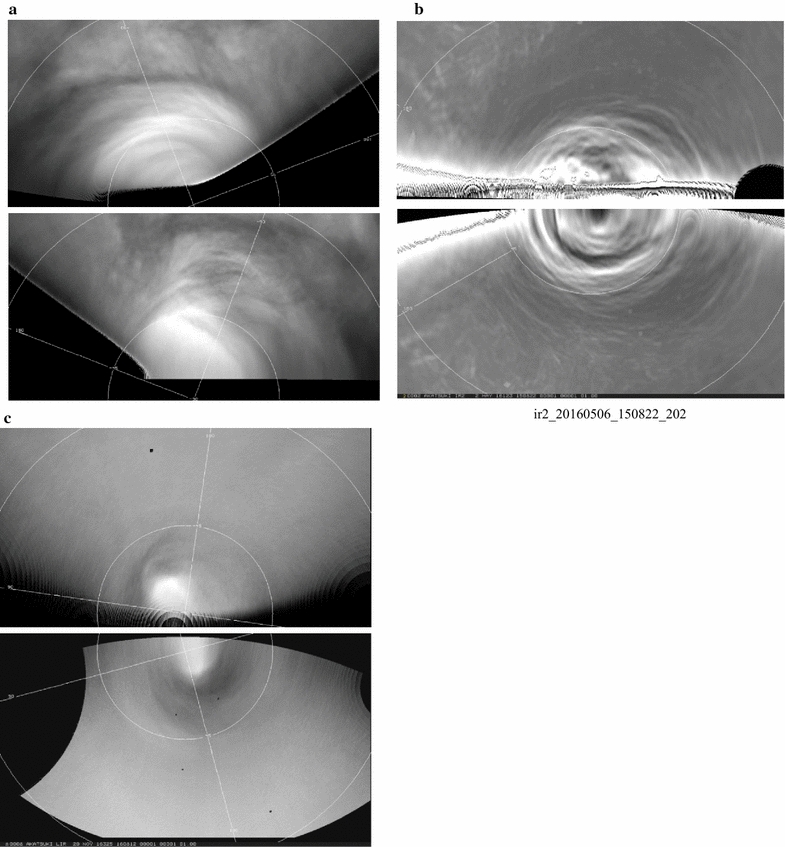
**a** Cloud cover at the ultraviolet shows the same vortex morphology over both hemispheres. These polar stereographic views of northern (top) and southern hemisphere (bottom) on dayside are from a 365-nm images taken on May 17, 2016, on orbit 15 of Akatsuki. Brightness normalization has been applied using Minnaert law. **b** North (bottom) and south (top) hemispheres in polar stereographic projection of a 2.02-µm contrast filtered image with overlaid latitude and longitude grid. The calibrated image was first subjected to a contrast filter to bring out the local structures and then projected. The pattern at higher latitudes resembles the spiral arms of the vortex seen at ultraviolet, but the inclination to latitude circles appears slightly different. Also, a lot more structure is seen in the contrast filtered versions closer to the poles. **c** Polar stereographic view of the southern hemisphere (top) from the LIR camera on September 16, 2016 (06:47:47 UT), and of the northern hemisphere (bottom) imaged on November 11, 2016 (16:08:12 UT). The respective pole is in the center and the periphery marks the equator in both of the images. Higher brightness temperatures near both poles are consistent with lower cloud tops as also seen in the 2.02-µm data. Faint arcs in both images are believed to be signatures of the spiral bands seen in the ultraviolet images as a result of the vortex organization of the circulation. The cold collar is clearly visible as a region of lower brightness temperatures in the northern hemisphere, but its appearance is not so distinct in the southern hemisphere indicating that the circulation plays a significant part in the cloud cover structure

One morphological feature that spans both the day and night cloud cover of Venus is the hemispheric global vortices in each hemisphere, rotating asymmetrically about each pole. First discovered from Mariner 10 images taken in February 1974 (Suomi and Limaye 1978), the vortex organization of the cloud level circulation has been seen in Pioneer Venus (Limaye 1985), Galileo (Peralta et al. 2007) and Venus Express (Limaye et al. 2009) data and hence, now can be assumed to be a quasi-permanent state of the global circulation. The VIRTIS experiment (Piccioni et al. 2007b) with its wide spectral coverage from ultraviolet to NIR was able to capture the complete day and night portions of the southern vortex and also reveal the intricacies of the core region on the nightside (Luz et al. 2011). Recently Muto and Imamura (2017) have further explored the oval shape of the vortex core region from VMC images. Akatsuki’s near-equatorial orbit does not provide an over the pole view of Venus, and thus, the complete vortex side cannot be seen in any day or night images and can be seen only in a space–time composite. Figure 20 presents a view of the vortex organization as obtained from Akatsuki observations at three wavelengths. An example is shown in Fig. 20a by using only two 365-nm images separated by ~ 2 days with one image rotated by 180° to show the correspondence of the spiral features. The central oval-shaped core region which is somewhat depressed in altitude can be seen. Figure 20b shows the finer structure in the north and south hemisphere vortices from 2.02-µm observations. The fine-scale structure could be due to variations in cloud altitudes if the absorption from CO_2_ is considered. This is consistent with the warmer brightness temperatures from the LIR data shown in Fig. 20c. The thermal infrared also shows the core region is warmer and also exhibits the vortex morphology (Fig. 20b). Images taken through 2-µm filters from IR2 suggest that the vortex structure persists in the middle cloud layer seen on the nightside, consistent with the VIRTIS observations. Generally the equatorward edge of the vortex over the poles is seen in all of the LIR images, depicting its temporal evolution due to the vortex dynamics (Garate-Lopez et al. 2013, 2015; Limaye et al. 2009; Piccioni et al. 2007a).

### The Y feature

The Y feature and its numerous shape variations observed in the ultraviolet images of Venus are seen in the Akatsuki images on the dayside quasi-periodically, but are conspicuously absent in the nighttime images taken at 1.74, 2.26 and 2.32 µm. It is also not seen at all in the thermal infrared wavelength images obtained from the LIR camera on the Akatsuki orbiter (discussed later). This suggests some limits on the vertical extent of the Y feature. This recurring feature was first noticed in Earth-based images and has been interpreted to appear as a result of the distortion of an equatorial wave as it propagates within a latitudinal varying zonal wind differing from a solid body rotation (Peralta et al. 2015). To some extent, the appearance or perception of the Y-shaped feature appears to be related to the relative spatial resolution of the images—it is far easier to observe this feature in lower spatial resolution images than in high-resolution images. This is due to the scale dependence of the contrasts seen in the Venus cloud cover. The contrasts on small spatial scales (≪ 100 km) are very low when compared to the contrasts seen at larger scales (≫ 100 km), as can be seen from images shown in Figs. 4, 5, 6 and 7. The lack of detection of the Y feature in the LIR data suggests little brightness temperature difference between the bright arms and the stem of the feature and the neighboring region seen at short wavelengths, which is a puzzle. It is worth noting that narrow band 11.34-µm images from the Subaru telescope (Sato et al. 2013, 2014, 2017) do occasionally show bow-like features that suggest the presence of the Y feature seen on the dayside. One explanation may be that the contrast producing absorber(s) are deeper than the emitting layer of the clouds and masked by the broad weighting function of the LIR camera. It is possible that additional analysis after removal of limb darkening may reveal a signature of the Y feature.

## Summary

The four imaging cameras on Akatsuki are revealing the dynamic evolution of the cloud features at the uppermost level of the clouds on the dayside and at deeper levels on the nightside. The appearance of Venus in the near-infrared wavelengths is very different from its dayside appearance, suggesting some vertical differences in the cloud properties. On the nightside, the morphologies of the deeper features appear disconnected from those seen on the dayside at the cloud tops. Whether these differences are related to local time variations or due to the layered nature of the clouds (Knollenberg and Hunten 1980) is not known. The nightside images show new features on the nightside, including curved features, suggesting generation of local vorticity which is very puzzling. The diversity of features seen in the mid- to lower clouds suggests a mixture of unknown dynamics as well as compositional differences that must be responsible for the differences in the appearance of the features. There also appears to be a subtle wavelength dependence of contrasts on spatial scale. The impression is that while on the dayside, the features seen from 283 nm to 2.02 µm are somewhat similar, the contrast magnitudes and spatial scales are different.

The cloud patterns at 365 nm seen in the Akatsuki images are similar to those seen from previous missions. The 283-nm images show features similar to those at 365 nm much of the time with lower contrasts, but occasionally show some different patterns. The longer wavelength images taken on the dayside show patterns resembling those observed at shorter wavelengths, but the details are very much muted. On the nightside, Akatsuki observations from the IR2 camera reveal new aspects of the cloud structure not seen in VIRTIS images—mesoscale vortices, long ribbons or waves of widths from 200 to 500 km with varying wavelengths of 800 to 1200 km or longer. Surprisingly, many wispy or string-like features similar to cirrus clouds on Earth are also seen on the nightside at low and midlatitudes which correspond to low opacity regions, indicating higher radiances from the atmosphere due to warmer temperatures. It is a mystery as to how such features develop.

There is considerable uncertainty in the level at which the features seen in the IR2 nightside images can be attributed from different studies (Barstow et al. 2012; Hueso et al. 2015), but generally it is believed that the level is somewhere between the base of the clouds and at least a scale height below the cloud tops and perhaps at 54 km, close to the level at which the VeGa 1 and VeGa 2 balloons traveled from midnight to somewhat beyond the morning terminator (Sagdeev et al. 1990) near the equator. Intermittent, periodic tracking by the very long baseline interferometry (VLBI) of the two balloons show a very zonal trajectory, devoid of significant meridional motions. The puzzle is to reconcile their relatively smooth zonal motion with the dynamics, as suggested by the morphologies seen in nighttime IR2 images—the VeGa balloons moved fairly smoothly and zonally at about 6.5° away from the equator in the north and south hemispheres over most of their 48-h trajectory, yet the IR2 nightside images show a rather chaotic, dynamical situation. The VeGa balloons did at times experience some large vertical accelerations (Blamont et al. 1986), and some were later found to be correlated with topography (Young et al. 1987), while convective overturning is also a possible cause. The standing gravity waves seen in LIR and UVI (283-nm) images are absent on the nightside. At the thermal wavelengths, standing waves and the core region of the vortices situated over the northern and southern poles are the most prominent features. Global brightness temperature contrasts are low (< ~ 5 K). Analysis of the Akatsuki data from all cameras is continuing, and these and future investigations should give us a better idea of the variety of different processes occurring in the atmosphere of Venus on both the day and night sides.

Our main conclusions are:

The vertical structure of the deep Venus clouds is complex and we do not know the precise altitude of the contrasts on the day- or nightsides. From remote sensing, we are limited to sampling the cloud-top region on the dayside and the deeper levels below the cloud tops on the nightside. Differences between the short and long wavelengths on the dayside attest to the presence of some larger particles near the cloud tops where discrete features can be seen in the longer wavelengths. The morphologies seen on day and night hemispheres are very different. It is not possible to know whether these vertical structure differences are due to local time effects or a global pattern, unlike the terrestrial occurrence. On Earth, clouds form due to phase change effects, whereas on Venus the clouds exist due to complex chemistry involving trace species. These differences are rooted in the differing makeup of the clouds on the two planets.

It is not well understood why the contrasts peak at 365 nm on the dayside and near 2.3 µm on the nightside. This spectral range is within the gap between the CO_2_, water vapor and other gaseous absorption peaks. Composition of the cloud particles, however, is not very well known, including their nature other than containing varying concentrations of sulfuric acid—particulate, microorganisms and shape. Photochemistry, ambient temperatures and/or dynamics may all be responsible for the appearance of Venus at these wavelengths in particular.

The boundaries between bright and dark regions from pole to pole as seen at different wavelengths do not appear to be at the same latitudes. For example, there is a clear boundary in the morphology patterns at midlatitudes at all wavelengths except at the thermal infrared (8–12 µm) where the boundary is between 60° and 70° latitude. The LIR data indicate brightness temperature changes across latitudes of no more than 10–15 K, and the 2.02-µm images do reveal some altitude differences due to different CO_2_ absorption at polar latitudes.

Regional dynamics must be involved in creating variations in trace gas abundances, which must control the cloud opacity and reflectivity variations. How these variations come about cannot be determined due to little information about their sources as most trace species are affected by cloud composition. The differences in the day- and nightside reaction rates of the numerous possible chemical reactions in the cloud layer may be somehow responsible.

In order to understand the wavelength differences in the appearance of Venus, we need more information and a better understanding of the role of trace species, condensation nuclei and cloud chemistry under the environmental conditions in the cloud layers as well as knowing the nature of the aerosols—organic or inorganic. To obtain these measurements, instrumented mobile platforms capable of sampling different regions of the Venus cloud layer over day and night are needed. Longer period coverage of the night hemisphere of Venus between 1.74 and 2.3 µm is essential in this respect. While most of the global cloud cover modeling studies based on the reference model (Kliore et al. 1985) appear to explain many of the previously observed features (Barstow et al. 2012; Haus et al. 2017; Lee et al. 2016), the nightside images suggest noticeable horizontal differences in the cloud structure. Insufficient data for many of the observed new features are a serious problem for understanding their origins, and new observations are very much needed. Indeed, it would be very welcome to have near continuous coverage of the day and night hemispheres of Venus in selected wavelengths from new missions.

The Akatsuki orbiter continues to function normally, and along with the UVI, LIR, LAC and radio occultations are collecting data. The IR1 and IR2 team are continuing efforts to restore data collection. The nominal mission will last until December 2018, and a proposal to extend the funding to continue mission operations for an additional 3 years is pending with JAXA.

## Abbreviations

CEB: circum equatorial belt
IR1: infrared (1 µm) camera on Akatsuki
IR2: infrared (2 µm) camera on Akatsuki
IRTF: Infrared Telescope Facility on Mauna Kea, Hawaii
LAC: Lightning and Airglow Camera on Akatsuki spacecraft
LIR: long-wave infrared imager on Akatsuki spacecraft
MDIS: mercury dual imaging system on MESSENGER spacecraft
NASA: National Aeronautics and Space Administration
nm: nano meter
OIR: Orbiter Infrared Radiometer on Pioneer Venus Orbiter
PVO: Pioneer Venus Orbiter
SSI: solid-state imaging camera on Galileo spacecraft
UVI: ultraviolet imager
VIRTIS: visible infrared thermal imaging spectrometer on Venus Express
VMC: Venus Monitoring Camera on Venus Express
VOI: Venus orbit insertion
